# Cryoelectrospun elastin-alginate scaffolds as potential cell delivery vehicles for mesenchymal stromal cell therapy

**DOI:** 10.1038/s41598-025-03822-x

**Published:** 2025-06-05

**Authors:** Pujhitha Ramesh, Rafael Pena, Jennifer M. Morrissey, Nicholas Moskwa, Kate Tubbesing, Xulang Zhang, Deirdre Nelson, James Castracane, Alexander Khmaladze, Susan T. Sharfstein, Melinda Larsen, Yubing Xie

**Affiliations:** 1 Department of Nanoscale Science and Engineering, University at Albany, State University of New York, 257 Fuller Road, Albany, NY 12203 USA; 2 Department of Biological Sciences, University at Albany, State University of New York, 1400 Washington Avenue, Albany, NY 12222 USA; 3 The RNA Institute, University at Albany, State University of New York, 1400 Washington Avenue, Albany, NY 12222 USA; 4 Department of Physics, University at Albany, State University of New York, 1400 Washington Avenue, Albany, NY 12222 USA; 5 Present Address: Regeneron Pharmaceuticals, 81 Columbia Turnpike, Rensselaer, NY 12144 USA; 6 Present Address: The Jackson Laboratory of Genomic Medicine, 10 Discovery Drive, Farmington, CT 06032 USA; 7 Present Address: Neural Stem Cell Institute, 150 New Scotland Ave, Albany, NY 12208 USA

**Keywords:** Cryoelectrospinning, Elastin-alginate scaffold, MSC delivery, Fibrosis, Salivary gland, *In vivo* implantation, Biomedical engineering, Mesenchymal stem cells, Nanobiotechnology

## Abstract

Salivary gland fibrosis results in salivary hypofunction whose current treatments are palliative. Mesenchymal stem/stromal cells (MSCs) are anti-fibrotic and anti-inflammatory and provide a promising alternative to treat fibrosis, but face translational challenges due to poor tissue targeting and retention. Scaffolds could facilitate targeted MSC delivery and improve MSC retention. Here, we demonstrated the feasibility of using stromal extracellular matrix (ECM)-mimicking elastin-alginate cryoelectrospun scaffolds (CES) for MSC delivery. Using MSC-like primary embryonic salivary mesenchyme cells (SMSCs), we demonstrated that CES supported viable, healthy and non-fibrotic long-term stromal cell maintenance while suppressing the fibrotic phenotype in myofibroblasts. We established an in vitro fibrosis model by coculturing SMSCs and myofibroblasts on CES and validated that SMSCs on CES imparted anti-fibrotic effects on myofibroblasts; however, these effects were suppressed by TGF-β1. In vivo orthotopic implantation of CES and SMSC-CES constructs showed that CES were biocompatible, non-inflammatory, and non-fibrotic, however, they did not remediate fibrosis. Further, supplementation of anti-fibrotic FGF2 to the in vitro fibrosis model demonstrated partial rescue of the anti-fibrotic effects of SMSC-CES constructs on myofibroblasts stimulated by TGF-β1 signaling. These studies set the premise for multi-pronged therapeutic approaches and validate CES as a potential stromal cell delivery vehicle in regenerative medicine applications.

**Supplementary Information:**

The online version contains supplementary material available at 10.1038/s41598-025-03822-x.

## Introduction

Mesenchymal stem/stromal cell (MSC) therapy is promising for treating cancer, fibrosis, degenerative disorders, and fighting COVID-19. MSC therapy has gained significant traction in remediating degeneration of the brain, heart, liver, kidney, salivary gland, etc., through regenerative, immunomodulatory, and anti-fibrotic mechanisms^1^. Fibrosis and cancer are both substantial disease burdens, contributing to 45% ^2^ and 21% ^3^ of US deaths, respectively. Many degenerative diseases (e.g., arthritis, osteoporosis, neurodegenerative diseases) can currently be managed but not cured. MSC therapy purports a potential non-palliative treatment for many of these diseases with possibility for lifetime cure.

Fibrosis is an organ-impairing disease caused by chronic inflammation and characterized by aberrant accumulation of extracellular matrix (ECM). Fibrosis is associated with high mortality due to impairment of many organ systems, including liver, kidney, lung, salivary gland, heart, cornea and skin^4–11^. Salivary gland fibrosis can be caused by the autoimmune disorder Sjögren’s Disease, diabetes mellitus, or radiation therapy in head and neck cancer patients^12,13^. These conditions lead to salivary hypofunction because the fibrotic stroma inhibits secretory salivary epithelium. Current treatments for salivary hypofunction include topical mucosal lubricants, saliva substitutes, sugar-free lozenges, saliva stimulators, acupuncture, or transcutaneous electrostimulation, which are all palliative and often produce severe side effects^14,15^. MSC therapy has shown improved salivary gland function in patients with radiation therapy-induced salivary hypofunction by remediation of fibrosis, increased serous tissue composition, and improvement of saliva output^16^. While MSC therapy has demonstrated tremendous potential in improving organ function, current MSC delivery strategies suffer from poor homing, low target-specific engraftment rates and transient therapeutic effects.

Current therapeutic MSC delivery strategies include systemic, intramuscular, transepidermal or direct injection into tissues/organs^17,18^. Systemic delivery of MSCs through either intravenous or intraarterial routes is the most used MSC delivery strategy due to ease of implementation. However, the clinical translation of this therapeutic strategy has faced some severe limitations, including lack of control over the biodistribution of MSCs delivered, poor MSC retention for more than 7 days, poor engraftment of MSCs delivered, emboli formation in organs with microcapillaries, and poor targeting of organs and homing to sites of injury/disease^18,19^. Further, animal studies have demonstrated that the biodistribution of MSCs in organs is dependent on the age of the recipient^19^, with poor MSC retention in vivo in aged populations, the main target of MSC therapy for remediation of degenerative diseases. Thus, the full potency of MSC therapy remains unrealized, lacking a delivery strategy to maximize retention of MSCs post-implantation.

Scaffold-based cell delivery may improve targeted delivery, engraftment rate, and MSC retention duration^20^, boosting treatment potency^21^. Further, mechanical cues derived from scaffolds can modulate the secretory and regenerative potential of MSCs^22,23^. However, the choice of scaffolds for MSC delivery is crucial since inappropriate mechanical cues from scaffolds can activate MSCs to transdifferentiate into myofibroblasts^24^, losing their pro-regenerative and anti-inflammatory properties. MSCs typically reside in a soft ECM in vivo^25^, and therefore, scaffolds that emulate native soft tissue ECM could potentially maintain the MSC phenotype. Further, matrices that mimic physiological ECM could prove therapeutic by modulating the behavior of the delivered MSCs and tissue-resident fibrotic cells, maximizing therapeutic efficiency, as observed in functional motor recovery in spinal cord-injury patients^26^.

We have previously bioengineered elastin-alginate cryoelectrospun scaffolds (CES) mimicking the soft tissue properties of decellularized salivary gland matrix (DSG). We compared the physical and rheological properties of CES and DSG and demonstrated that both matrices exhibit similar porous honeycomb topography with ~ 20 μm pores, indentation modulus of ~ 100 Pa, relaxation half time of 100–200 s and support immortalized stromal cell growth^27,28^. In this work, we examined the potential of using CES for MSC cell delivery, targeting fibrosis remediation. Using MSC-like primary embryonic day 16 (E16) salivary mesenchyme cells (SMSCs) as our MSC source, we compared the ability of CES to support a healthy stromal cell phenotype vs. decellularized salivary gland (DSG) matrices, which closely recapitulate the in vivo microenvironment^29^, and Matrigel, the conventional vehicle for salivary gland organoid culture^30^. In this comparison, we examined the expression of healthy stromal phenotypic markers and myofibroblast markers using immunocytochemistry and confocal imaging, followed by image quantification (Figure S1), a widely accepted approach for protein expression quantification. We also compared SMSCs on CES with DSG for their ability to remediate the fibrotic phenotype of myofibroblasts in vitro. Finally, we evaluated the biocompatibility and anti-fibrotic effects of the SMSC-CES constructs by orthotopic implantation studies, followed by in vitro evaluation of FGF2’s ability to improve stromal phenotype and enhance the anti-fibrotic effects of the SMSC-CES constructs.

## Results

### Cryoelectrospun scaffolds allow viable 3D stromal cell growth comparable to decellularized salivary gland matrices

**Fig. 1:**
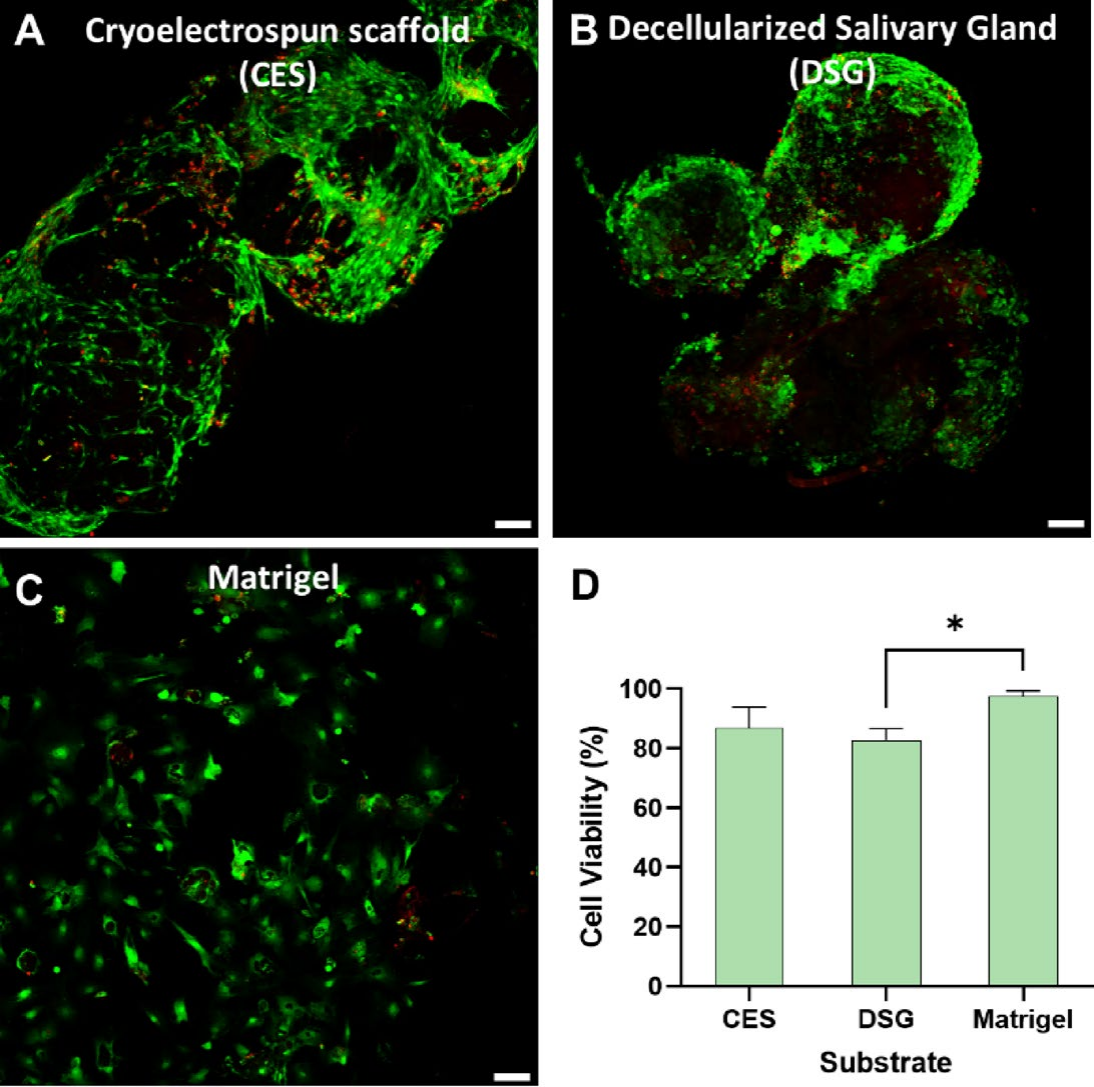
Cryoelectrospun scaffolds (CES) support viable 3D cell growth for up to 7 days. Confocal microscopy images of LIVE/DEAD stained SMSCs grown in (**A**) CES (*n* = 3), (**B**) decellularized salivary gland matrices (DSG, *n* = 3), and (**C**) Matrigel (*n* = 3). Green, live cells; Red, dead cells; Yellow-Orange, dying cells that stained both green and red. Scale bar = 100 μm. (**D**) Image analysis-based quantification of cell viability after 7 days of 3D cell growth in a 24 well plate reveals that cells in CES have viability levels comparable to DSG and approaching that of Matrigel. Statistical analysis was performed by one-way ANOVA with Tukey’s multiple comparisons test. *p < 0.05.

To test the feasibility of using elastin-alginate cryoelectrospun scaffolds (CES) as a stromal cell delivery vehicle, we examined the scaffolds’ ability to retain stromal cell viability. We chose SMSCs since in prior work we have shown that they retain stemness properties^31^, expressing CD105 (in a subset), CD140a/PDGFRα and CD140b/PDGFRβ markers (Figure S2), and are available in the large quantities required for our experiments. Cell seeding and culture on scaffolds were optimized by evaluating the effect of static versus rotary culture on cell attachment in the first 24 h following cell seeding (Figure S3), as well as the effect of the size of the culture well plate (24 vs. 48-well plate) on longer-term cell viability in 7-day cultures (Figure S4). We grew SMSCs for 7 days on elastin-alginate CES and compared cell viability with DSG for physiological relevance and with Matrigel, a standardized in vitro cell culture matrix for stem cells and organoids. Cell viability on CES and DSG was considerably high at 87 ± 6% and 82 ± 3% respectively (Fig. 1). Cells grown in Matrigel showed the highest viability at 98 ± 1%, possibly due to better nutrient transfer to cell sheets than to the 3D cell clusters observed in CES and DSG (Fig. 1).

### Cryoelectrospun scaffolds promote preferential healthy stromal phenotype in SMSC

**Fig. 2:**
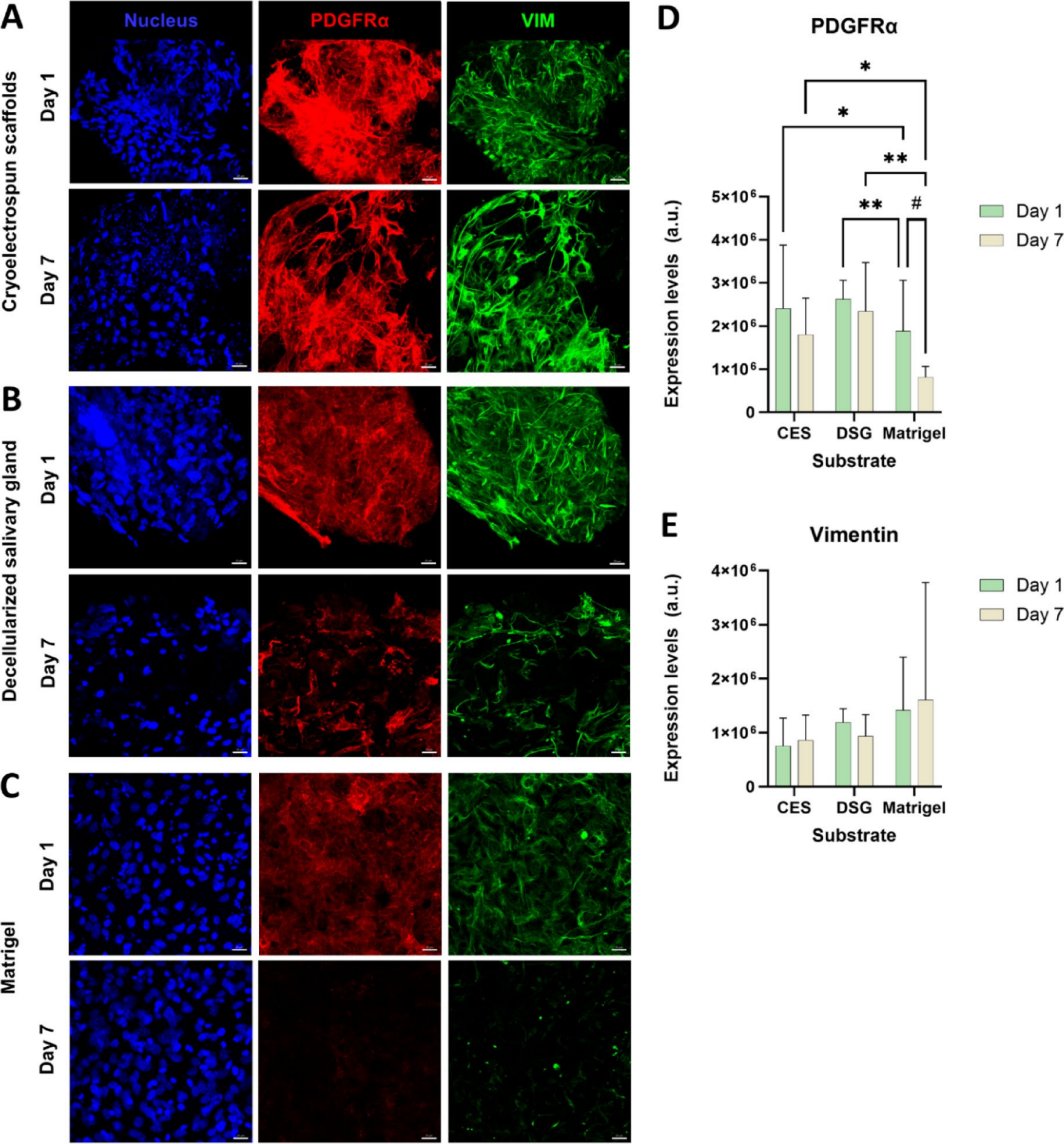
Cryoelectrospun scaffolds (CES) promote the maintenance of healthy stromal marker protein expression in SMSCs. Confocal microscopy images showing expression of PDGFRα (red) and vimentin (green) in SMSCs grown in (**A**) CES, (**B**) decellularized salivary gland matrices (DSG), and (**C**) Matrigel for 1 and 7 days. Scale bar = 20 μm. Quantitative image analysis of (**D**) PDGFRα and (**E**) vimentin confirm that CES support stromal marker expression in SMSCs for up to 7 days. PDGFRα expression in CES is comparable to DSG and better than Matrigel on day 1 and day 7, respectively. Vimentin expression is similar in CES, DSG and Matrigel confirming the maintenance of fibroblast phenotype. Statistical analysis was performed by two-way ANOVA with uncorrected Fisher’s LSD for multiple comparisons, for comparisons between substrates (p values denoted by *); For comparisons between day 1 and day 7 data points within the same substrate, multiple unpaired t tests without correction for multiple comparisons were performed (p values denoted by ^#^). *Day 1*: CES, *n* = 10. DSG, *n* = 3. Matrigel, *n* = 11. *Day 7*: CES, *n* = 8. DSG, *n* = 8. Matrigel, *n* = 10. *,^#^*p* < 0.05, **p < 0.01.

To test the feasibility of using CES for MSC delivery, we examined their ability to maintain a healthy stromal phenotype compared with DSG and Matrigel. We grew SMSCs for up to 7 days on the scaffolds and evaluated stromal health by expression of stromal markers, PDGFRα, and vimentin using immunostaining and confocal imaging. PDGFRα is a PDGF family membrane receptor expressed in salivary gland stromal cells during organogenesis^32^. Our previous work^33^ demonstrated that PDGFRα^+^ stromal cell subpopulations regulate secretory epithelial phenotype. Vimentin is a standard mesenchymal cytoskeletal marker up-regulated in fibrosis^34^. The stromal cells had a characteristic spread-out fibroblast morphology in CES, DSG, and Matrigel after 7 days in culture, with PDGFRα and vimentin expression (Fig. 2A-C). Quantitative protein expression analysis from confocal images revealed that PDGFRα protein expression in SMSCs grown in CES and DSG were comparable and similarly maintained on day 7 compared to day 1 (Fig. 2A, B, D). In contrast, PDGFRα expression in Matrigel drastically decreased from day 1 to day 7 and was lower than in both CES and DSG (Fig. 2A-D). Average vimentin protein expression levels remained similar in all experimental groups without significant changes (Fig. 2A-C, E), confirming the maintenance of fibroblast phenotype, but expression levels varied dramatically (> 100% standard deviation) in the Matrigel Day 7 group (Fig. 2E). PDGFRα and vimentin gene expression analysis (Figure S5 A, B) confirmed similar expression trends at the transcriptional level with minor variations.

### Cryoelectrospun scaffolds restrict myofibroblast marker expression in SMSCs in comparison to decellularized salivary glands

**Fig. 3:**
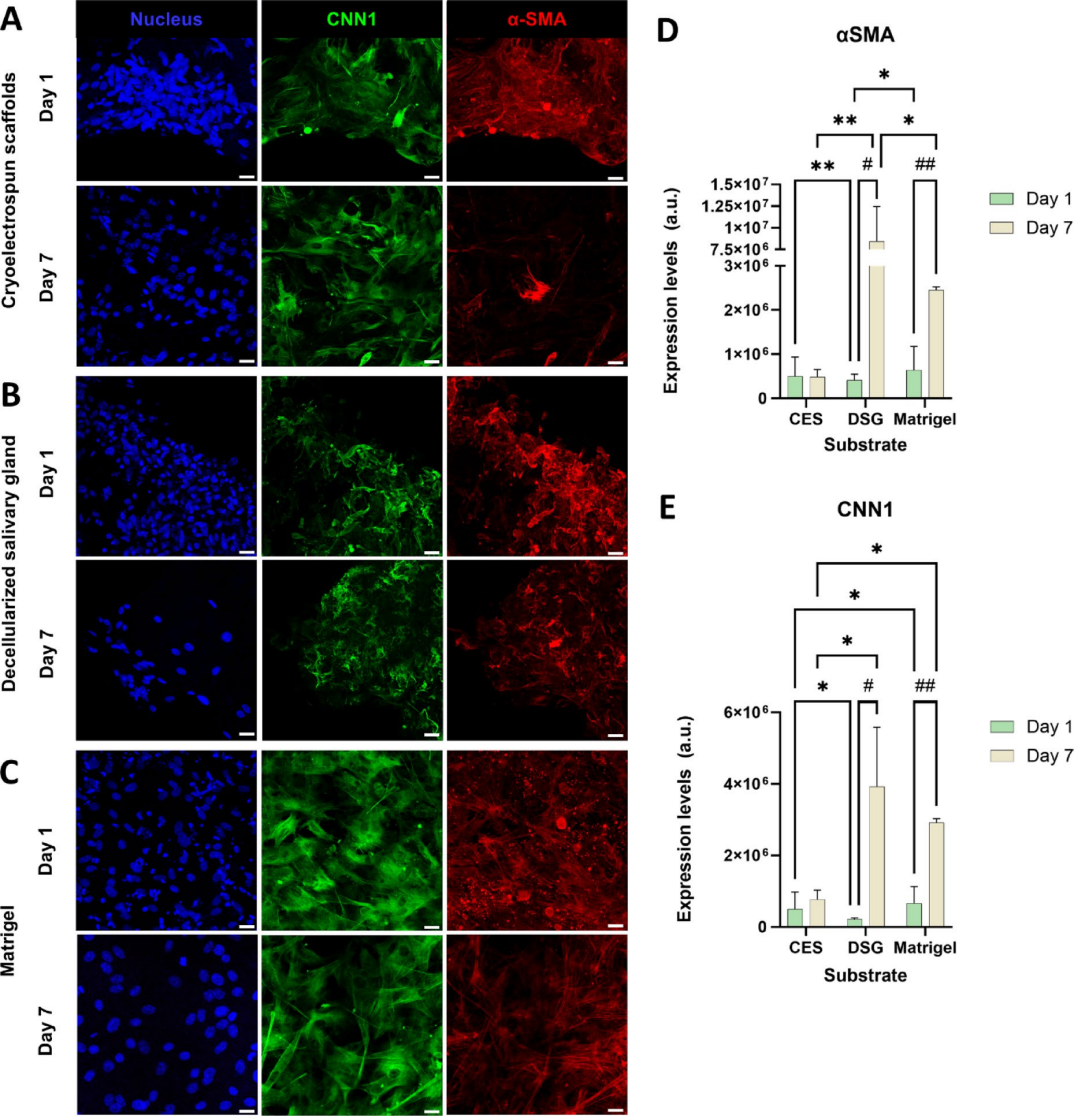
Cryoelectrospun scaffolds (CES) demonstrate time-dependant suppression of fibrotic marker expression in SMSCs more effectively than decellularized salivary gland matrices (DSG). Confocal microscopy images showing expression of CNN1 (green) and α-SMA (red) in SMSCs grown in (**A**) CES, (**B**) DSG, and (**C**) Matrigel for 7 days. Scale bar = 20 μm. Quantitative image analysis of (**D**) α-SMA and (**E**) CNN1 confirms that CES restrict myofibroblast marker expression in SMSCs for up to 7 days much better than both DSG and Matrigel. Statistical analysis was performed by two-way ANOVA with uncorrected Fisher’s LSD for multiple comparisons, for comparisons between substrates (p values denoted by *). For comparisons between day 1 and day 7 data points within the same substrate, multiple unpaired t tests without correction for multiple comparisons were performed (p values denoted by ^#^). *Day 1*: CES, *n* = 12. DSG, *n* = 3. Matrigel, *n* = 2. *Day 7*: CES, *n* = 6. DSG, *n* = 3. Matrigel, *n* = 3.*,^#^*p* < 0.05, **,^##^*p* < 0.01.

To determine whether cryoelectrospun scaffolds could suppress fibrotic activity in healthy stromal cells or prevent myofibroblast-like differentiation, we grew SMSCs for up to 7 days in CES, DSG, or Matrigel, and examined the expression of myofibroblast markers, α-SMA and CNN1, by immunostaining, confocal imaging and quantitative image analysis. α-SMA and CNN1 are cytoskeletal proteins up-regulated in fibrotic conditions, indicating myofibroblast activity^35^. Transcriptional analysis of α-SMA (Figure S5 C) in CES, DSG, and Matrigel revealed that α-SMA expression in CES was comparable to that in DSG and significantly lower than in Matrigel on both day 1 and day 7 (*p* = 0.0089, Figure S5 C). α-SMA and CNN1 protein expression did not change in CES (Fig. 3A, D, E) from day 1 to day 7; however, in DSG (Fig. 3B, D, E) and Matrigel (Fig. 3C, D, E), α-SMA (*p* = 0.025 and *p* = 0.008, respectively) and CNN1 (*p* = 0.018 and *p* = 0.003, respectively) expression levels increased significantly by day 7 and were much higher than in CES.

### Cryoelectrospun scaffolds suppress fibrotic activity in myofibroblasts

**Fig. 4:**
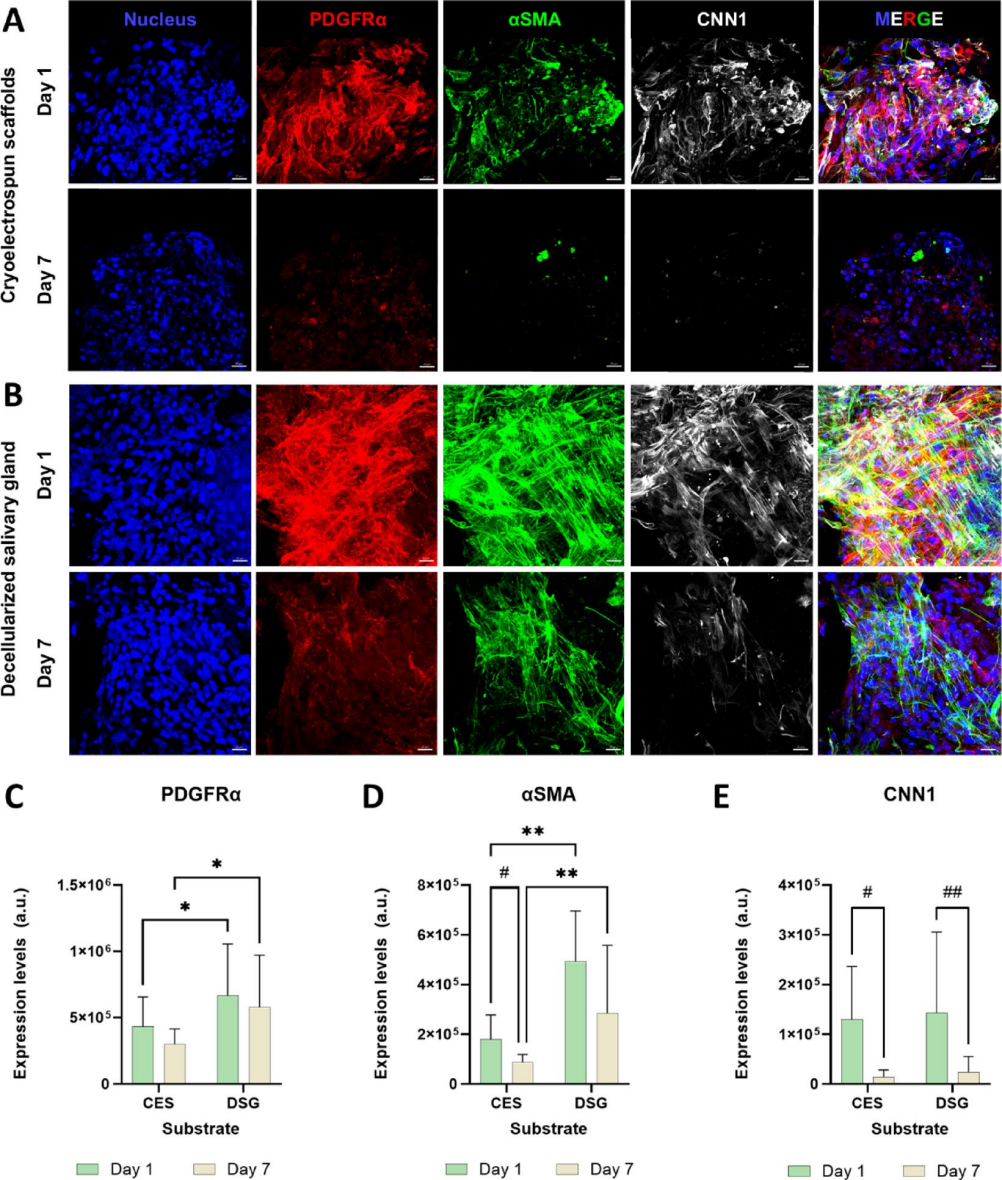
Cryoelectrospun scaffolds (CES) suppress fibrotic phenotype in myofibroblasts cultured in them over a period of 7 days. Confocal microscopy images showing expression of PDGFRα (red), α-SMA (green), CNN1 (white) in myofibroblasts grown in (**A**) CES and (**B**) decellularized salivary gland matrices (DSG) for 1 and 7 days. Scale bar = 20 μm. Quantitative image analysis of (**C**) PDGFRα, (**D**) α-SMA and (**E**) CNN1 confirm that CES maintain PDGFRα expression albeit at a lower level than DSG, suppress α-SMA expression more effectively than DSG and suppress CNN1 expression comparably to DSG over a period of 7 days, demonstrating that CES has anti-fibrotic properties. Statistical analysis was performed by two-way ANOVA with uncorrected Fisher’s LSD for multiple comparisons, for comparisons between substrates (p values denoted by *). For comparisons between day 1 and day 7 data points within the same substrate, statistical analysis was performed by multiple unpaired t tests without correction for multiple comparisons (p values denoted by ^#^). *Day 1*: CES, *n* = 7. DSG, *n* = 10. *Day 7*: CES, *n* = 6. DSG, *n* = 16. *,^#^*p* < 0.05, **,^##^*p* < 0.01.

Soft scaffolds of low stiffness (~ 3 kPa) have been shown to reverse the fibrotic phenotype in activated hepatic stellate cells in liver fibrosis^36^. Previously, we established that CES have a low stiffness of ~ 120 Pa and a relaxation half time of 100–200 s, which simulates the viscoelasticity of DSG^27^. To examine the potential of soft tissue-like CES, to suppress fibrotic activity in myofibroblasts, myofibroblasts were cultured in CES for 7 days (Fig. 4A), and compared with myofibroblasts grown in DSG (Fig. 4B). Both CES and DSG suppressed myofibroblast fibrotic activity as demonstrated by the significant reduction in α-SMA expression in CES (*p* = 0.048) and CNN1 expression in CES (*p* = 0.023) and DSG (*p* = 0.008), after 7 days in culture compared with day 1 expression levels (Fig. 4A, B, D, E). PDGFRα expression levels in myofibroblasts also decreased from day 1 to day 7 in both CES (Fig. 4A, C) and DSG (Fig. 4B, C). However, PDGFRα expression levels in myofibroblasts grown in CES were lower than in DSG on day 1 and day 7 (Fig. 4C). Further, the expression level of α-SMA in myofibroblasts grown in CES was significantly lower than that in DSG (*p* = 0.0014), while CNN1 expression in CES was comparable to DSG, consistent with the anti-fibrotic effects of CES.

### SMSCs on cryoelectrospun scaffolds suppress fibrotic activity in myofibroblasts

**Fig. 5:**
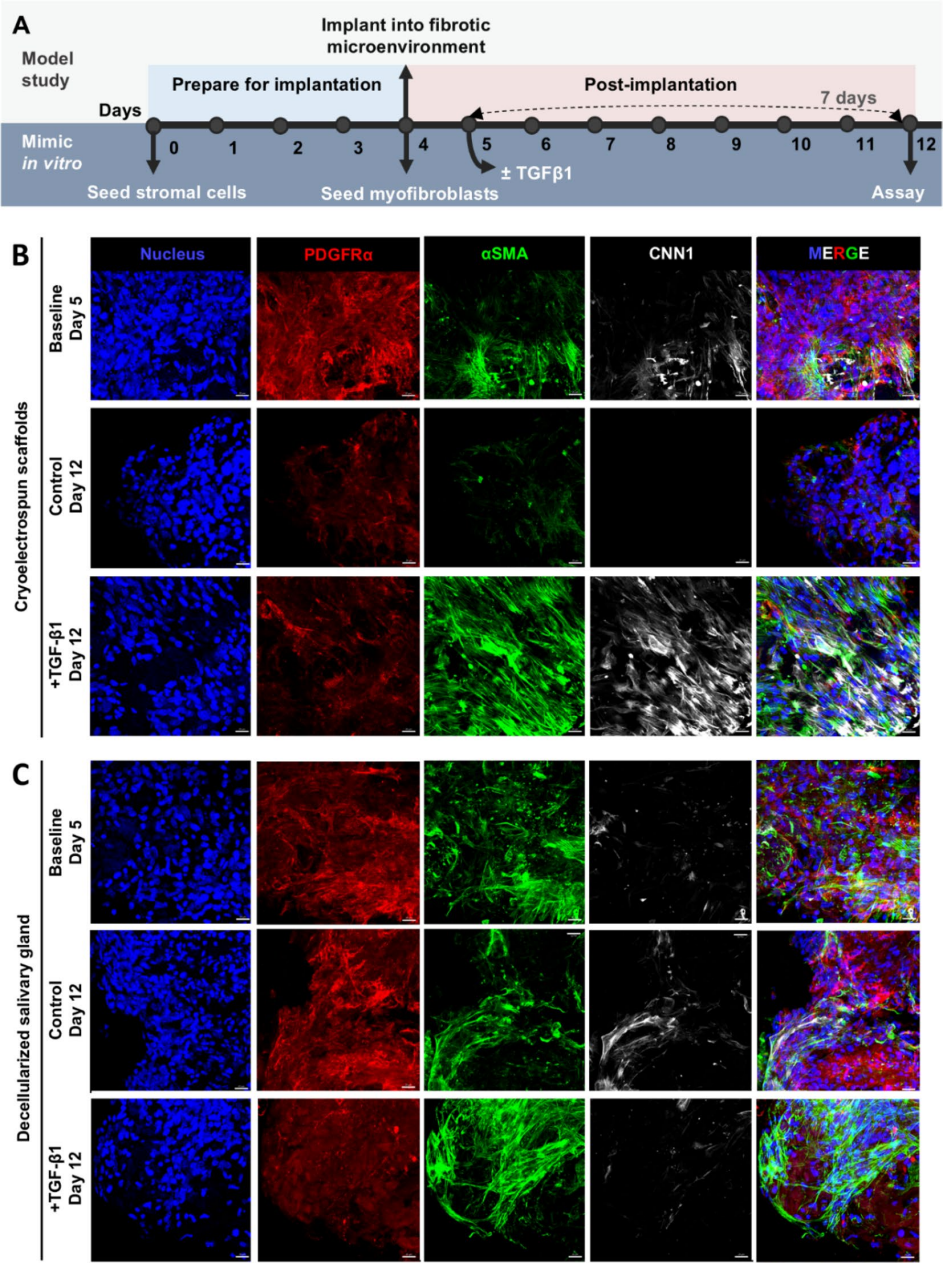

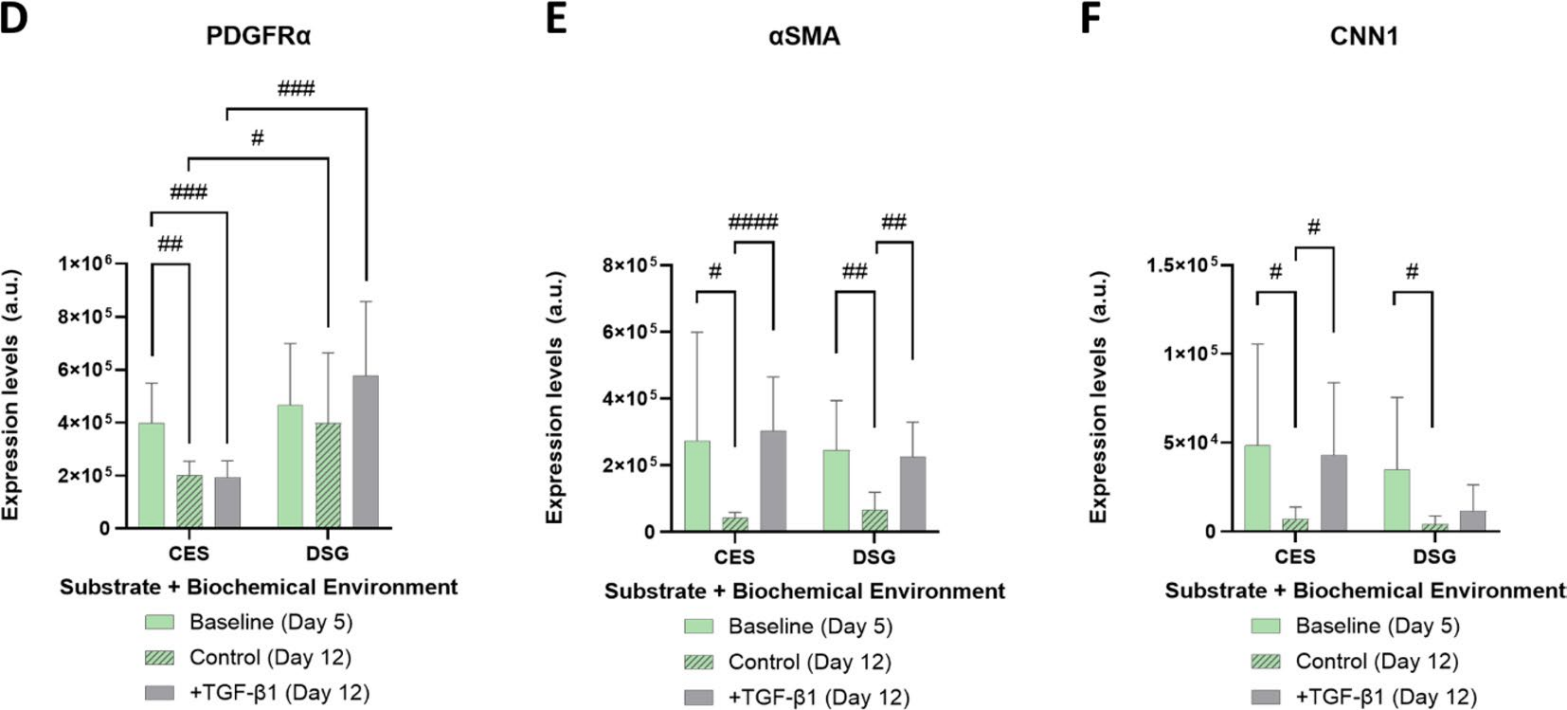
SMSCs in cryoelectrospun scaffolds (CES) impart anti-fibrotic effects on myofibroblasts over time comparably to decellularized salivary gland matrices (DSG) but fall short in a TGF-β1-induced fibrotic environment. (**A**) Schematic of in vitro experiment design to simulate implantation of SMSCs in CES or DSG into an in vivo fibrotic environment, containing myofibroblasts and TGF-β1. Confocal microscopy images of myofibroblasts and SMSCs after one day of coculture on day 5 (Baseline Day 5), and after 7 days of coculture in the absence (Control Day 12) or presence of TGF-β1 (+ TGF-β1 Day 12) on (**B**) CES and (**C**) DSG, immunostained for PDGFRα (red), α-SMA (green) and CNN1 (white). Scale bar = 20 μm. Fluorescence intensity-based quantitative image analysis of (**D**) PDGFRα, (**E**) α-SMA and (**F**) CNN1 expression. Statistical analysis was performed by multiple unpaired t tests without correction for multiple comparisons. *Baseline Day 5*: CES, *n* = 10. DSG, *n* = 8. *Control Day 12*: CES, *n* = 10. DSG, *n* = 9. *+TGF-β1 Day 12*: CES, *n* = 10. DSG, *n* = 6. ^#^*p* < 0.05, ^##^*p* < 0.01,^###^*p* < 0.001,^####^*p* < 0.0001.

To determine if SMSCs cultured in CES can suppress fibrotic activity in myofibroblasts, we established an in vitro fibrosis model to mimic implantation of MSCs into an in vivo fibrotic environment, recapitulated by myofibroblasts with and without TGF-β1, a well-established pro-fibrotic cytokine^37^ (Fig. 5A). First, we primed SMSCs in CES and DSG for 4 days, allowing cells to attach and acclimate to the 3D culture environment (observed via cell spreading and expression of characteristic stromal markers), before introducing myofibroblasts, to simulate implantation into a fibrotic microenvironment. We chose day 4 as the timepoint to mimic implantation since cell spreading of SMSCs on CES improved after 4 days in culture (Figure S6). Myofibroblasts were seeded onto SMSC-CES constructs or cell-DSG constructs, and cells were cocultured for one day allowing cells to attach and acclimate. Some cell-scaffold samples were collected on day 5 (Baseline), while others were cocultured for 7 additional days with or without TGF-β1 (+ TGF-β1 and Control, Day 12), simulating the biological cues in a fibrotic microenvironment.

SMSCs suppressed fibrotic activity in myofibroblasts (Fig. 5B, C Baseline vs. Control panels) in CES and DSG as evidenced by the significant reduction of α-SMA (*p* = 0.038 and *p* = 0.004, respectively, Fig. 5E) and CNN1 protein expression (*p* = 0.035 and *p* = 0.038, respectively, Fig. 5F), compared to their respective baseline levels after 7 days in co-culture. PDGFRα expression significantly decreased in co-cultures in CES (*p* = 0.0011) but not in DSG (Fig. 5D). α-SMA and CNN1 expression in SMSC-myofibroblast coculture were not significantly different in CES vs. DSG at day 5 or day 12 timepoints.

In a TGF-β1-induced fibrotic microenvironment, α-SMA and CNN1 expression in SMSC-myofibroblast cocultures remained at day 5 baseline levels and were significantly higher than in the control group in CES (*p* = 0.00008 and *p* = 0.013, respectively); whereas in DSG, α-SMA (*p* = 0.0016) was significantly higher and CNN1 was considerably higher (Fig. 5B, C, E, F). After 7 days in culture, PDGFRα expression levels in SMSC-myofibroblast cocultures in CES and DSG were not significantly different between groups, either with or without TGF-β1. Also, after 7 days in culture, PDGFRα expression levels in the SMSC-myofibroblast cocultures were significantly higher in DSG compared to CES (Control, *p* = 0.033; +TGF-β1, *p* = 0.0008), irrespective of TGF-β1 presence. The maintenance of α-SMA and CNN1 expression in CES and DSG in the presence of TGF-β1 at levels comparable to day 5 baseline levels, even after 7 days, and the significant decrease in α-SMA and CNN1 expression in the absence of TGF-β1 in CES and DSG.

### In vivo implantation of cryoelectrospun scaffolds embedded with and without SMSCs does not elicit an inflammatory or fibrotic response

After confirming the potential of CES to maintain healthy stromal phenotype in SMSCs, and their anti-fibrotic activity in vitro, we conducted orthotopic implantation studies to determine whether CES can facilitate local delivery of cell-based therapeutics to fibrotic submandibular glands (SMG) in vivo. We tested the in vivo biocompatibility of CES by implanting CES with and without SMSCs into mice that had undergone SMG partial resection injury to model fibrosis and evaluated inflammatory responses in non-fibrotic and local fibrotic regions. Glands were collected from all animals 14 days after resection for these initial studies as there is a persistent fibrotic response in one region of the gland after 14 days^38^ as shown in Figure S9.

**Fig. 6:**
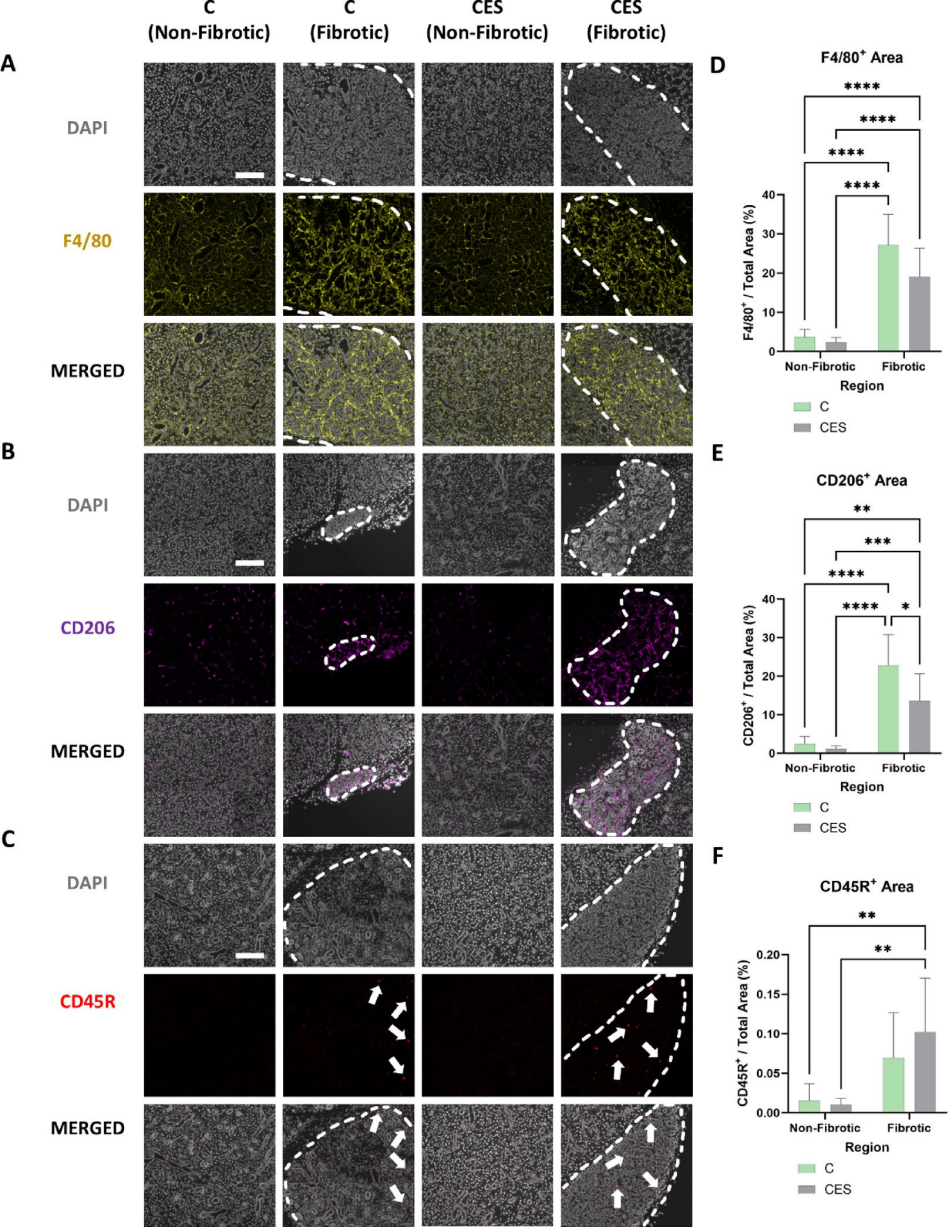
Implantation of cryoelectrospun scaffolds (CES) did not induce an inflammatory response. Representative images of non-fibrotic and local fibrotic regions in resected salivary glands (**C**) and resected salivary glands implanted with CES stained with DAPI (gray). Expression of (**A**) the macrophage marker F4/80 (yellow), (**B**) M2 macrophage marker CD206 (purple), and (**C**) B cell marker CD45R (red). Quantification of the area of (**D**) F4/80, (**E**) CD206, and (**F**) CD45R in both the non-fibrotic and fibrotic regions normalized to the total area of the ROI, with *N* = 7 mice (**C**) and 8 mice (CES). Statistical analysis was performed by two-way ANOVA with Bonferroni correction. *p < 0.05, **p < 0.01 and ***p < 0.001. Scale bars = 50 μm.

Immune cell infiltration into the fibrotic and non-fibrotic regions was evaluated by staining for pan-macrophage marker F4/80 (both M1 pro-inflammatory and M2 anti-inflammatory macrophages), M2 macrophages marker CD206, and lymphocytic B-cell marker CD45R. We previously detected a significant increase in macrophages in the local fibrotic region of SMGs following partial gland resection, with a primary M2 response observed through increase in macrophages expressing CD206^38^. Therefore, cryopreserved SMG sections were subjected to immunohistochemistry analysis of these markers to determine if CES implantation induces an inflammatory response beyond the local fibrotic response. As expected, we saw an increase in the macrophage marker F4/80 (Fig. 6A) and CD206 (Fig. 6B) following resection in the local fibrotic region but not in healthy, non-fibrotic regions of the gland. The lymphocytic B-cell marker CD45R revealed few lymphocytes in the local fibrotic region following gland resection (Fig. 6C). However, there was no significant change in F4/80 nor CD45R in the local fibrotic or the healthy non-fibrotic regions of the gland following implantation of CES. CD206, however, significantly decreased in the local fibrotic region following implantation of CES (*p* = 0.0159) when compared to the local fibrotic region of the resected glands alone (Fig. 6D-F). These results indicate that following partial resection, the implantation of CES does not significantly change the infiltration of macrophages into the fibrotic region; however, M2 macrophages may decrease. Overall, orthotopic implantation of CES scaffolds did not induce immune cell infiltration.

**Fig. 7:**
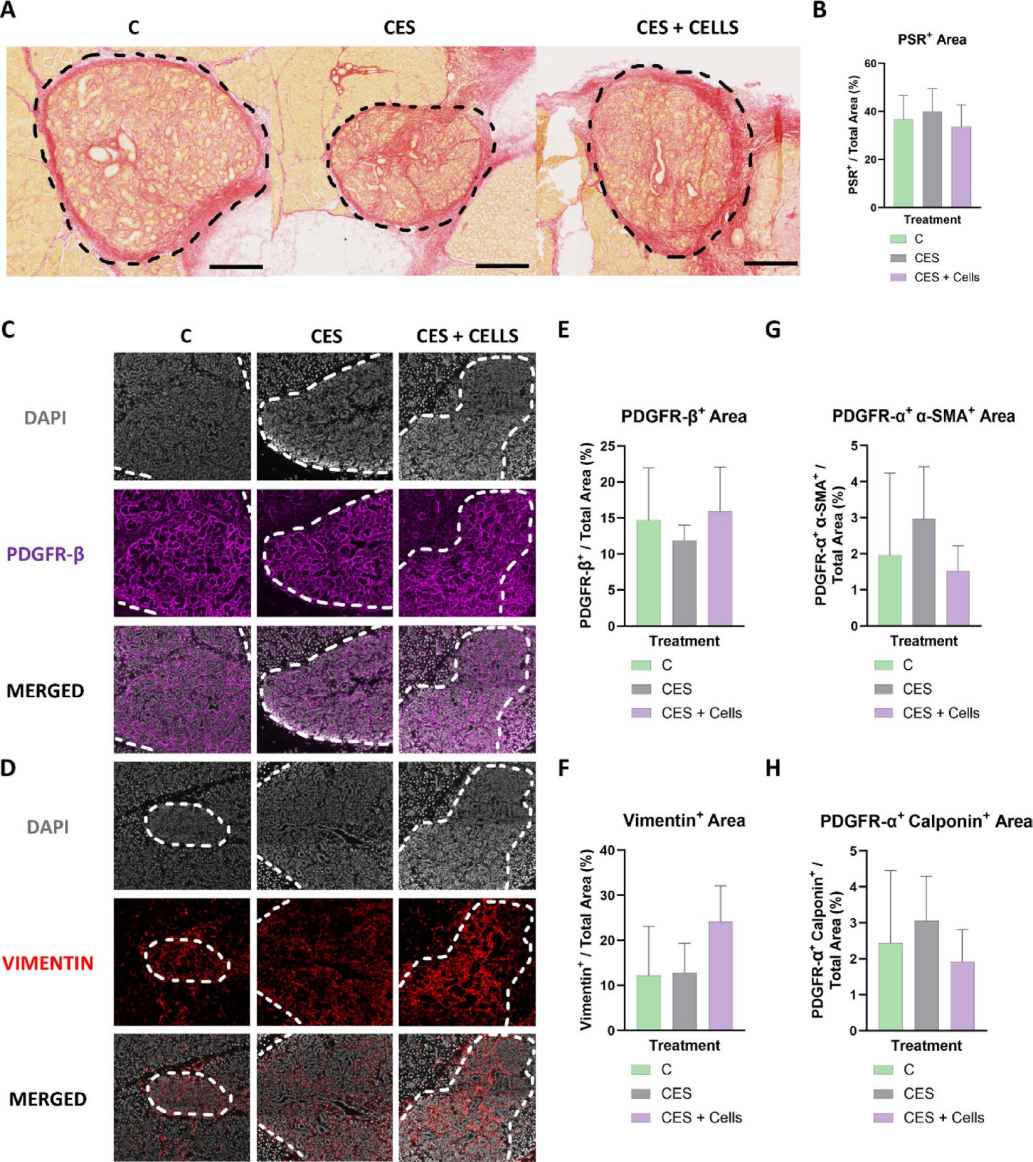
Implantation of cryoelectrospun scaffolds (CES) with or without SMSCs did not significantly promote or reduce fibrosis in the local fibrotic region. (**A**) Representative images of local fibrotic regions of resected salivary glands (**C**) and resected salivary glands implanted with CES alone (CES) or with SMSCs (CES + Cells) stained with PSR. (**B**) Quantification of PSR^+^ area normalized to the total area of the local fibrotic region shows no significant changes, with *N* = 7 mice (**C**), 8 mice (CES), and 10 mice (CES + Cells). (**C**) Representative images of local fibrotic regions of resected salivary glands (**C**) and resected salivary glands implanted with CES alone (CES) or with SMSCs (CES + Cells) stained with DAPI (gray) and PDGFRβ (purple) and (**D**) vimentin (red). (**E**) Quantification of PDGFRβ and (**F**) vimentin normalized to the total area of the local fibrotic region shows no significant change. (**G**) Quantification of PDGFRα co-expressing α-SMA or (**H**) Calponin (CNN1) normalized to the total area of the local fibrotic region shows no significant change, with *N* = 5 mice (**C**), 7 mice (CES), and 9 mice (CES + Cells). Statistical analysis was performed by two-way ANOVA with Bonferroni correction. *p < 0.05, **p < 0.01 and ***p < 0.001. Scale bars = 50 μm.

**Fig. 8:**
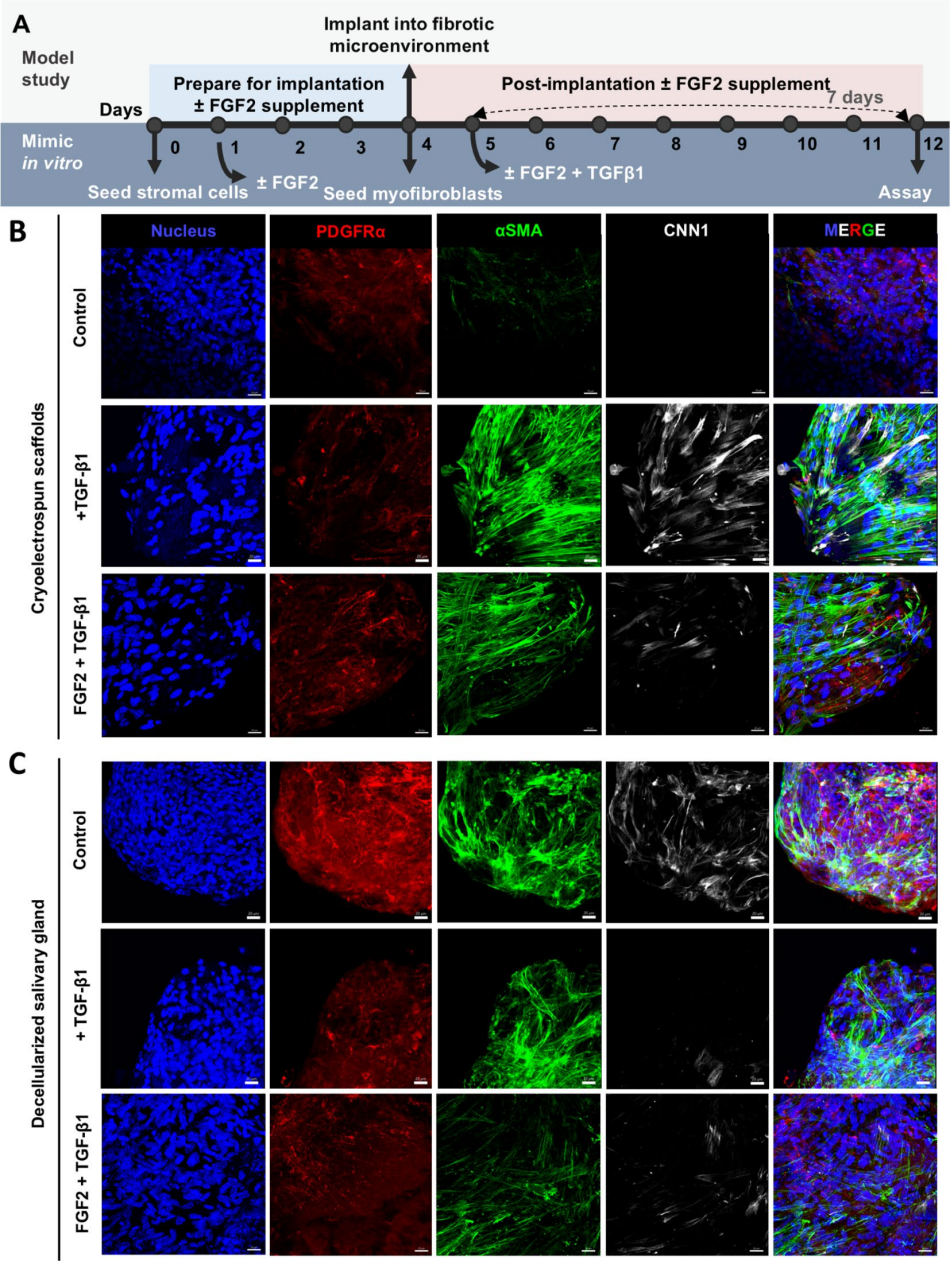

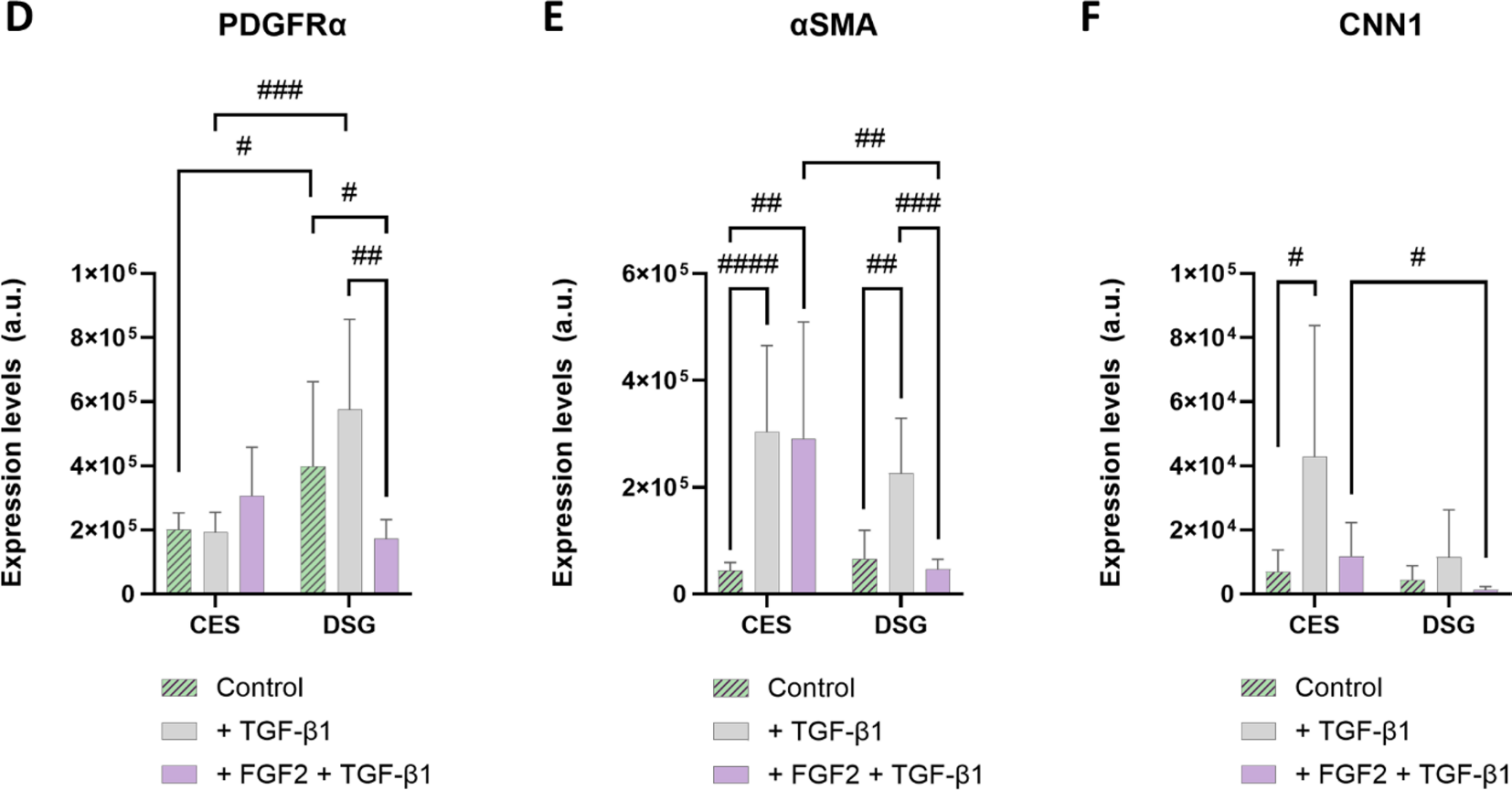
FGF2 partially rescues the anti-fibrotic effects of SMSCs in cryoelectrospun scaffolds (CES) that were suppressed by TGF-β1. (**A**) Schematic of in vitro experiment designed to mimic implantation into a fibrotic environment and interaction of SMSCs + FGF2 with myofibroblasts and TGF-β1, assayed on day 12. Confocal microscopy images of myofibroblasts and SMSCs after seven days of coculture on day 12 in the absence of both FGF2 and TGF-β1 (Control), in the presence of TGF-β1 (+ TGF-β1), and in the presence of both FGF2 and TGF-β1 (+ FGF2 + TGF-β1) on (**B**) CES and (**C**) DSG, immunostained for PDGFRα (red), α-SMA (green) and CNN1 (white). Scale bar = 20 μm. Fluorescence intensity-based quantitative analysis of (**D**) PDGFRα, (**E**) α-SMA and (**F**) CNN1 expression. Statistical analysis was performed by multiple unpaired t tests without correction for multiple comparisons. *Control*: CES, *n* = 10. DSG, *n* = 9. *+TGF-β1*: CES, *n* = 10. DSG, *n* = 6. *+ FGF2 + TGF-β1*: CES, *n* = 6. DSG, *n* = 8. ^#^*p* < 0.05, ^##^*p* < 0.01,^###^*p* < 0.001.

We previously observed a significant increase in ECM deposition in the local fibrotic region of partially resected salivary glands, which persisted for up to 56 days, as shown through trichrome staining^38^. Therefore, we performed picrosirius red (PSR) staining, which stains Collagen I in red, on slides and assessed whether CES-mediated local delivery of SMSCs could remediate the local fibrosis after gland resection. PSR staining and quantification revealed no significant change in the local fibrotic region in the presence of CES scaffold implantations without or with SMSCs relative to resection alone (Fig. 7A-B). Expression of PDGFRβ has been reported to increase upon injury^39^, and we previously reported that vimentin is increased in the local fibrotic region^38^. Immunohistochemistry staining and quantification of PDGFRβ (Fig. 7C) and vimentin (Fig. 7D) revealed that CES alone and SMSC-CES constructs did not alter these stromal markers after gland resection in the fibrotic region (Fig. 7E-F). Immunohistochemistry staining and stain quantification for PDGFRα co-expressing myofibroblast markers α-SMA or CNN1 proteins showed that CES alone and SMSC-CES constructs did not alter myofibroblast markers in the fibrotic region (Fig. 8G-H) 14 days after implantation. These results necessitate further optimization of the MSC-CES constructs to assess potential remediation of fibrosis.

### FGF2 partially rescues the anti-fibrotic effects of SMSC-cryoelectrospun scaffold constructs on myofibroblasts

The suppression of the anti-fibrotic effects of CES and SMSCs on CES by TGF-β1 in vitro and the lack of fibrosis reduction in vivo 14 days post-implantation prompted us to evaluate additional modifications to the implant system to enhance the anti-fibrotic effects of the cell-CES constructs. FGF2 is well recognized for its potent anti-fibrotic properties^40^ and its ability to improve the therapeutic potential of MSCs^41^. We previously showed that FGF2 regulates PDGFRα + salivary mesenchyme cells to stimulate secretory epithelial phenotype differentiation^33,42^. Here, we observed that FGF2 supplementation promoted a healthy stromal phenotype and suppressed α-SMA gene expression in SMSCs over 7 days (Figure S7 A-C). To determine if FGF2 can augment the anti-fibrotic properties of SMSCs in CES in suppressing the fibrotic phenotype in myofibroblasts, especially in the presence of TGF-β1, we evaluated its activity in the in vitro fibrosis model described above. Briefly, we primed SMSCs in CES and DSG for 4 days, with or without FGF2 stimulation, followed by myofibroblast seeding on day 4 and TGF-β1 stimulation with or without FGF2 from days 5–12 (Fig. 8A).

On Day 12, after 7 days of TGF-β1 exposure, α-SMA and CNN1 protein expression in the SMSC-myofibroblast cocultures increased significantly in CES (*p* = 0.00008 and *p* = 0.013, respectively) compared to the TGF-β1-free control samples, whereas in DSG, α-SMA increased significantly (*p* = 0.0016) and CNN1 increased considerably (Fig. 8B, C, E, F). In this context, PDGFRα expression remained relatively unaffected in both CES and DSG (Fig. 8B-D). FGF2 pretreatment of SMSCs on the CES and DSG, followed by sustained stimulation by FGF2 after myofibroblast and TGF-β1 introduction, reduced CNN1 expression substantially in both CES and DSG and significantly reduced α-SMA (*p* = 0.0003) expression in DSG, comparable to control group expression levels (Fig. 8B, C, E, F). PDGFRα protein expression levels increased in CES whereas they decreased significantly in DSG (*p* = 0.0034) in the FGF2 and TGF-β1-stimulated group compared to both TGF-β1 only and control groups (Fig. 8B-D). These results demonstrate suppression of fibrotic activity in myofibroblasts by SMSCs on both CES and DSG when stimulated with FGF2, despite TGF-β1 signaling. The overall expression levels of α-SMA and CNN1 were significantly lower in DSG than in CES when treated with FGF2 and TGF-β1 (*p* = 0.0076 and *p* = 0.015, respectively, Fig. 8E, F).

Treatment of SMSC-myofibroblast cocultures on CES and DSG with FGF2 alone from days 5–12 (Figure S8 A) confirmed that FGF2 potentiates the antifibrotic effects of SMSCs on CES and DSG through suppressed expression of α-SMA (*p* = 0.032 and *p* = 0.009, respectively) and CNN1 (*p* = 0.0058 in DSG) proteins (Figure S8 C, D) and increased expression of PDGFRα (*p* = 0.0019) in CES (Figure S8B), and that TGF-β1 addition eclipses the effects of FGF2 in both CES and DSG (Figure S8E-G). Nonetheless, upon stimulation by FGF2 and TGF-β1, PDGFRα (*p* = 0.013), α-SMA and CNN1 (*p* = 0.033) levels substantially reduced in DSG while only CNN1 (*p* = 0.0075) significantly reduced in CES compared to their respective baseline day 5 levels (just after myofibroblast seeding and before FGF2 and TGF-β1 stimulation, Figure S8E-G). These results reiterate that FGF2 has a potent anti-fibrotic effect in DSG but a milder anti-fibrotic effect in CES.

## Discussion

Stromal cells including MSCs typically reside in a soft ECM and are sensitive to the matrix stiffness, cell adhesion site density, and ECM protein composition^24,43^. Here, we established that engineered CES were similar to DSG and better than Matrigel in maintaining stromal phenotype. As Matrigel is a basement membrane extract derived from the Engelbreth-Holm-Swarm mouse tumor, it is heterogenous and ill-defined, and not suitable for cell delivery to treat diseases^44^. To retain optimal cell function and therapeutic efficiency, cell delivery vehicles should maintain cell viability and permit 3D cell-cell and cell-substrate interactions. Both CES and DSG showed > 80% cell viability, 3D clustering and characteristic fibroblast morphology for SMSCs. Unlike DSG and Matrigel, CES supported SMSCs phenotype through the expression of healthy stromal markers, PDGFRα and vimentin (Fig. 2), and suppression of myofibroblast markers, α-SMA and CNN1 after 7 days in culture. Hence, even though the bulk viscoelastic properties of CES and DSG are similar^27^, the material composition of CES and DSG induce different cell responses. Overall, CES demonstrated superior retention of the homeostatic stromal phenotype for cell delivery applications under standard cell culture conditions and provide an alternative to Matrigel for in vitro cell culture and future in vivo delivery. Future studies will need to delineate the specific characteristics of CES that facilitate this response.

Beyond preventing increased expression of myofibroblast markers, α-SMA and CNN1 in SMSCs, CES further suppressed the fibrotic phenotype in myofibroblasts, potentially de-differentiating them into fibroblast-like cells. Soft matrices have reduced the fibrotic phenotype of liver and lung myofibroblasts^45–47^, and the low stiffness of elastin-alginate CES (~ 100 Pa) might confer their anti-fibrotic properties. An in vitro analysis of suppression of myofibroblast fibrotic activity by SMSC-CES constructs in the presence or absence of TGF-β1 revealed that SMSCs could suppress fibrotic activity in the absence of TGF-β1 but its anti-fibrotic effects were overpowered by TGF-β1 in a fibrotic microenvironment. Although we previously showed that stromal cells derived from embryonic salivary glands promoted proacinar differentiation of epithelial cells^48^, and demonstrated here that SMSCs on CES suppressed fibrotic marker expression in myofibroblast cocultures in vitro, the SMSC-CES constructs did not demonstrate significant regression in fibrosis *in vivo.* Although mice treated with SMSC-CES constructs demonstrated a downward trend in α-SMA, CNN1 and PSR + stained regions, the anti-fibrotic effect is inconclusive. This result is consistent with the observations from our in vitro TGF-β1 stimulated fibrosis model and validates its functionality for evaluating the anti-fibrotic potential of scaffolds, cells, and cell-scaffold constructs. This result also highlights the limitation of this in vivo study where only a single dosage of SMSCs at a single treatment duration were assessed for anti-fibrotic effects. While our implantation study provides crucial insight into the biocompatibility of the scaffold evidenced by unaffected immune cell infiltration, and its translational potential, it also highlights the need to refine the therapeutic payload through extensive and comprehensive therapeutic efficacy studies in the future. One potential reason for the lack of anti-fibrotic effects in vivo by the CES, could be due to the harvest time of two-weeks post orthotopic implantation. We chose the two-week time point since our primary objective was to check if CES are biocompatible and non-inflammatory. However, a longer treatment of 12–16 weeks might be needed to evaluate the efficacy of MSC delivery effects on fibrosis, as in prior MSC therapy studies^49–51^. Alternatively, as the in vivo fibrotic niche is complex and tightly regulated by multiple fibrosis modulators^52,53^, a combination therapy strategy may be necessary to synergistically tackle the several pro-fibrotic factors.

To further boost the anti-fibrotic potential of SMSCs in CES, we tested FGF2, which both supports stromal anti-fibrotic activity and imparts anti-fibrotic effects of its own^40,41^. FGF2 suppressed α-SMA expression in SMSCs in vitro. However, FGF2 could only suppress fibrotic activity in myofibroblasts in the absence of TGF-β1, with only mild anti-fibrotic effects observed in a TGF-β1-induced fibrotic microenvironment. This is not surprising because in in vivo microenvironments TGF-β1 signaling is tightly regulated since TGF-β1 is latent and needs to be activated to become bioavailable; however, in in vitro cultures TGF-β1 supplementation makes it readily bioavailable, and unregulated TGF-β1 is a potent fibrosis stimulator^54,55^. SMSCs in DSG supplemented with FGF2 could more effectively suppress fibrotic activity in myofibroblasts both in the presence and absence of TGF-β1 compared to CES. DSG consists of the ECM of the homeostatic salivary gland, and biochemical and micro-mechanical cues from several ECM proteins might complement the anti-fibrotic effects of FGF2, facilitating synergistic modulation of TGF-β1 signaling. CES with its simplistic ECM composition might not have facilitated the complex ECM interactions with FGF2 and TGF-β1, warranting future work to alter the composition of the CES so that they more closely resemble the homeostatic ECM. Supplementing SMSC-CES constructs with additional anti-fibrotic modulators, such as TGF-β1 inhibitors and/or hyaluronic acid as part of the biomaterial^56,57^, as combination therapy strategies may fortify the anti-fibrotic properties of the SMSC-CES constructs in the presence of potent fibrosis mediators such as TGF-β1.

Furthermore, SMSCs may not be as potent as MSCs from other sources in regulating the microenvironment of myofibroblasts in the presence of pro-fibrotic stimulants. Administration of adipose or bone-marrow derived MSCs, which have remediated fibrosis in the liver^58^, and regenerated the structure of necrotic SMG tissue^59^, in CES will elucidate whether these MSCs grown in CES can retain their anti-fibrotic properties even in the presence of TGF-β1 in vitro and in vivo. Also, optimal MSC dosages and longer treatment durations may be needed to achieve therapeutic efficacy.

Overall, to achieve measurable anti-fibrotic outcomes in vivo, the MSC-CES implant will require iterative refinement in the future, including testing different dosages of MSCs delivered and alternative sources of MSCs, extending treatment durations for time course evaluation, evaluating combination therapies with additional fibrosis modulators, and employing different in vivo fibrosis models. Nevertheless, this work establishes a robust foundation for validating the potential of CES as a biocompatible therapeutic delivery platform and highlights the necessary optimization to maximize its therapeutic efficacy.

In summary, the impact of this work lies in that first, we developed a biocompatible, non-inflammatory, and non-fibrotic CES that demonstrates potential to function as a MSC delivery vehicle without compromising the therapeutic properties of the MSC payload. It serves as a therapeutic prototype that needs future optimization to translate as an effective anti-fibrotic therapy in vivo. Second, we validated the use of CES for in vitro fibrosis modeling and demonstrated potential as an impactful preclinical research tool that can reduce the burden on animal model research. CES’ comparable performance to DSG, confers them an advantage over DSG, since DSG are limited in supply, whereas CES has the potential for mass manufacturing. Furthermore, CES can be manufactured from recombinant elastin and non-immunogenic alginate biomaterials, and our in vivo implantation study indicated that mice implanted with CES did not suffer immune rejection. Ultimately, these studies highlight the potential of CES, MSCs, and other anti-fibrotic supplements, such as FGF2, to synergistically modulate a fibrotic environment for the remediation of fibrosis in salivary glands and other organs.

## Methods

### Animals

Mice used to source salivary glands and MSC-like primary E16 mesenchyme (SMSC) were either of the CD-1 strain from Charles River Laboratories (Wilmington, MA) or C57BL/6 strain from Jackson Laboratories (Bar Harbor, ME). For in vivo implantation studies, female C57BL6/J strain mice were used. All mice were maintained by the University at Albany animal facility. Five mice were housed per cage and kept in a 12-hour light/dark cycle. Mice were ear punched with unique identifiers. The care and handling of mice and all procedures, tissue and fluid collections were performed in accordance with the NIH Guide for the Care and Use of Laboratory Animals and ARRIVE guidelines. Animal protocols were approved by the Institutional Animal Care Use Committee (IACUC) at the University at Albany, SUNY.

### Isolation and cell culture of mouse primary E16 salivary mesenchyme

Primary mesenchyme was isolated from embryonic day 16 (E16) SMG dissected from timed-pregnant CD-1 female Mus musculus (Charles River Laboratories), as described previously^30,42^. To enrich the embryonic salivary gland for stromal cells with an MSC-like phenotype, E16 mouse embryos were harvested, and the SMGs were removed and reduced to a mixture of stromal cells using collagenase/hyaluronidase, dispase, and mechanical dissection. Gravity sedimentation was used to separate the SMSCs from the epithelial clusters.

The isolated SMSCs were cultured in DMEM/F12 medium (Cat. # 11039047, ThermoFisher Scientific, Carlsbad, CA) supplemented with 10% fetal bovine serum (FBS) (Cat. # 10082147, ThermoFisher Scientific) and 1% penicillin and streptomycin (PenStrep, 10,000 U/mL, ThermoFisher Scientific) and incubated in a 37 °C, 5% CO_2_ humidified incubator for 3–4 days until 90–95% confluence. The SMSCs were cultured for 1 passage before use. The medium was typically replaced every day.

### Myofibroblast induction and culture

Myofibroblasts were differentiated from SMSCs by subculturing them for 4 or 5 passages and then treating them with 5 ng/mL of TGF-β1 (Cat. # 240-B, R&D Systems, Minneapolis, MN) for 1 or 2 passages until exhibiting myofibroblast-like morphology^33^, determined by an increase in cell surface area and the number of filopodia.

### Scaffold preparation

Elastin-alginate cryoelectrospun scaffolds with honeycomb topography (CES) were fabricated by cryoelectrospinning a 1% elastin, 1.5% alginate, and 3% PEG-400kD solution in deionized water, as described previously^27^. Briefly, the viscous polymer solution was loaded into a 3 mL syringe and pumped out of a 25G needle at a constant flow rate of 10 µL/min. The needle voltage was maintained at 17 kV, and the needle tip-to-collector spacing at 15 cm, relative humidity at > 40%, and air temperature at < 2 °C for cryoelectrospinning for 1 h onto a 5 mm metallic probe array collector plate maintained at ~ −20 °C. The cryoelectrospun scaffolds were then lyophilized for 3 h and crosslinked by the coupling chemistry of 1-Ethyl-3-(3-dimethylaminopropyl)carbodiimide (EDC) and N-Hydroxysuccinimide (NHS) (1.48 mg EDC and 1.78 mg NHS per 100 µL of 95% ethanol per scaffold) by rocking the scaffolds at 45 rpm for 2 h, followed by a series of graded ethanol washes with 95, 70, 50, and 0% ethanol in the presence of 1.5% CaCl_2_ for 15 min each to wash away residual EDC and NHS, and simultaneously, ionically crosslink the alginate chains. The crosslinked scaffolds were frozen at −80 °C overnight and lyophilized again for 4 h. The lyophilized scaffolds were sterilized by UV irradiation for 1 h, followed by incubation in 70% ethanol for 30 min. The scaffolds were then washed in 0.9% NaCl with 50 mM CaCl_2_ for 10 min in two times. Finally, the scaffolds were incubated in DMEM F12 medium with 5% penicillin-streptomycin and 4% amphotericin-B overnight prior to cell seeding.

Decellularized salivary gland matrices (DSG) were obtained by decellularizing whole organs resected from adult female CD-1 or C57BL/6 mice, as described in our previous work^27^. The decellularized salivary gland was stabilized using forceps on the stage of a dissecting microscope and sectioned into equally sized pieces using a vibratome blade. These decellularized salivary gland matrices were stored at 4 °C in cell culture medium for up to 2 weeks before culture.

Matrigel scaffolds were prepared by mixing 5 µL each of Matrigel (Cat. # 356234, Corning Inc., Corning, NY) and cell suspension (10,000 cells/µL). 10 µL of the cell-Matrigel suspension solution was pipetted onto a 0.1 μm Nuclepore polycarbonate filter (Cat. # 0930051, Cytiva, Marlborough, MA). The filter was floated on cell culture medium in 50-mm glass-bottom dishes (Cat. # P50G-1.5–14 F, MatTek Corporation, Ashland, MA) and incubated at 37 °C in a humidified tissue culture incubator with 5% CO_2_.

UV-irradiated cryoelectrospun scaffolds, and decellularized salivary gland matrices were sterilized by soaking in 70% ethanol for 30 min, washed with 0.9% NaCl for 10 min, and then hydrated in cell culture medium with 5% penicillin-streptomycin and 4% amphotericin B (R&D Systems) overnight prior to cell seeding for culture.

### Stromal cell culture on scaffolds

SMSCs were seeded on the various scaffolds under rotary culture at 30 rpm for the first 24 h, as detailed in supplementary materials (Table S1). After 24 h, the cell-scaffold constructs, except those grown in Matrigel, were transferred into wells of a 24-well plate that were coated with Lipidure (AMSBIO, Cambridge, MA) to minimize cell adhesion to the well bottom and facilitate cell attachment to the scaffold. Cell-containing scaffolds were grown in 24-well plates in 300 µL media for improved oxygen-mass transfer and cell viability relative to a 48- or 96-well plate while incubating without rotary shaking. For experiments requiring treatment with FGF2 (Cat. # Z200015, Applied Biological Materials, Vancouver, CA) and/or TGF-β1, growth factors were added to the medium 1 day after cell seeding, at final concentrations of 100 ng/ml FGF2 and 5 ng/ml TGF-β1, respectively. Cell culture media were replenished daily by removing 150 µL of spent medium from each well and replenishing it with 200 µL of fresh medium to avoid nutrient depletion and retain some conditioned media. Cells were cultured on scaffolds for up to 7 days after growth factor addition.

### Co-culture of SMSCs and myofibroblasts on scaffolds

To mimic stromal cell delivery to a fibrotic microenvironment, 50,000 SMSCs were first seeded on the scaffold, as described in supplementary materials, and grown for 4 days as described above, mimicking preparation for scaffold-based cell delivery. Subsequently, 50,000 myofibroblasts were seeded, as described in supplementary materials (Table S1), and maintained in culture as described above, to mimic their interaction post-implantation. Cells were cultured on scaffolds in the absence or presence of growth factors (100 ng/ml FGF2 without or with 5 ng/ml TGF-β1) for up to 7 days after growth factor addition. Media were changed as described above.

### LIVE/DEAD assay

Cell-scaffold constructs were incubated with 2 µM calcein-AM and 4 µM ethidium homodimer (EthD-1) (Sigma-Aldrich) for 25 min at 37 °C, rinsed once in 0.9% NaCl-50 mM CaCl_2_ solution by gentle pipetting and imaged using a Leica SP5 confocal laser scanning microscope (Leica Microsystems, Mannheim, Germany) to reveal live cells in green and dead cells in red fluorescence. Quantitative analysis of live and dead cells was performed using ImageJ^60^, as described previously^27^ and detailed in supplementary materials.

### Immunocytochemistry and confocal imaging

Immunocytochemistry and confocal imaging were performed using antibodies (Table S2) as described in supplementary materials.

### qPCR analysis

SMSCs were grown in CES, DSG, or Matrigel with or without TGF-β1 and/or FGF2 for 1 or 7 days. Three or four samples of each scaffold type and experimental condition were pooled to extract enough RNA using an RNeasy Micro kit (Qiagen, Germantown, MD) for qPCR analysis with primers shown in Table S3, as detailed in supplementary materials.

### Fluorescence intensity quantification of 3D images using Imaris

Image-based protein quantification was performed in Imaris 9.6.1 (Oxford Instruments, Tubney Woods, Abingdon, UK), as shown in Figure S1 and described in supplementary materials.

### Partial sialoadenectomy surgery on salivary glands

At 12 weeks of age, a partial resection was performed on the mouse’s right SMG, where 40% of the left distal tip of the SMG was excised while the mice were under 2% isoflurane anesthesia. After implantation of scaffolds, where relevant, incisions were closed with two to five interrupted sutures. The mice were monitored for pain, distress, and weight changes, and buprenorphine (0.015 mg/mL) was administered post-operatively, subcutaneously as an analgesic. A fibrotic response was induced in a localized region in the glands, which was confirmed 14 days post-surgery.

### Implantation of cryoelectrospun scaffolds into injured salivary glands

At the time of resection, the mice were subjected to experimental treatment, receiving one of three treatments, with 10 mice used for each treatment. The first treatment was a negative control of resection alone (i.e., no subsequent treatment). In the second treatment, five CES scaffolds were implanted into the resected glands. The third treatment involved seeding the five scaffolds with SMSCs 3 days before implantation and then implanting five SMSC-CES constructs per resected gland. Briefly, to prepare the implant for each mouse, 5 × 10^5^ SMSCs (passage 1) were seeded on five CES scaffolds placed in one well of a round bottom 96-well plate pre-coated with Lipidure and maintained in rotary culture (30 rpm) for 24 h; After 24 h each implant (five CES scaffolds with SMSC) was transferred to individual wells in a 24-well plate and cultured. Five CES scaffolds (per mouse), either incubated in cell culture medium (CES control) or seeded with SMSCs and cultured for 3 days, were washed with sterile saline at 37 °C and implanted into the void left adjacent to the partially resected glands, in the mice allocated for the respective treatments (10 mice/treatment). The scaffolds were left in the mice for 2 weeks. Partial resection was performed on 10 additional mice that did not receive scaffolds for comparison. Animals were euthanized under carbon dioxide (CO_2_) 14 days post resection/implantation. Salivary glands were harvested. The weights of the SMG with the sublingual gland (SLG) attached and the total mouse weight were measured. Cryopreserved SMGs were sectioned. All subsequent sample processing, image capturing, and quantitative analysis were performed blinded with respect to the unique identifiers from the ear punches.

### In vivo tissue harvesting and preparation

14 days following surgical implantation, the salivary glands (both SMG and SLG) and the remaining implanted scaffolds were removed together and frozen indirectly over liquid nitrogen and stored at −80 °C before cryosectioning. Tissue preparation and cryosectioning were detailed in supplementary materials.

### Immunohistochemistry analysis of cryosectioned slides

Cryosectioned slides were subjected to immunohistochemistry analysis of inflammatory markers, mesenchymal or myofibroblastic markers using antibodies listed in Table S2 and detailed in supplementary materials.

### Picrosirius red (PSR) staining of cryosectioned slides

Slides were fixed in Bouin’s Fluid at room temperature overnight. Afterwards, the slides were placed in a fresh container of Bouin’s Fluid, and then incubated at 60 °C for one hour. Slides were dipped in tap water 10 times and then incubated in PSR for 15 min. The slides were then rinsed in running tap water for one minute and then incubated in picric acid for 40 min. Afterward, the slides were incubated in isopropanol for 5 min. The slides underwent a series of dehydration steps, starting off with dipping the slides in 100% ethanol five times twice, ten dips in a 1:1 ratio of xylene/ethanol, one minute xylene wash twice, and finally, a two-minute xylene wash. The slides were then mounted in non-aqueous Fisher Chemical™ Permount™ mounting media (Thermo Fisher Scientific). Fibrotic vs. non-fibrotic regions were differentiated as detailed in supplementary methods and Figure S10.

### Imaging and quantification

All sections stained with PSR were imaged via tiling to capture whole gland images. Both SMG and SLG were imaged. Sections stained with PSR were imaged on a Hamamatsu NanoZoomer, RS2.0 Slide Scanner. Images were viewed using the Hamamatsu NDP.view2 Image viewing software U12388-01. Sections stained with antibodies for immunohistochemistry analysis were imaged on a Zeiss Z1 Cell Observer widefield with an Axio712 mono camera (Carl Zeiss, LLC) using a Plan-Neofluar 10 × 0.50 Ph2 M27 objective using the same settings for each set of samples. Regions that contained folds, tears, or artifacts were excluded from quantification. Quantification was performed on sections from similar tissue depths using FIJI Image J as detailed in supplementary materials.

### Statistical analysis

Data are presented as mean ± standard deviation. All in vitro cell culture experiments and in vivo animal experiments were performed in triplicate unless otherwise indicated. Unless otherwise indicated, all image analyses were performed on at least 3 samples per experimental group. Data from in vitro cell culture experiments were analyzed by one-way ANOVA with Tukey’s multiple comparison test, or two-way ANOVA with select multiple comparisons followed by uncorrected Fisher’s LSD test for multiple comparisons and/or multiple unpaired parametric t-tests without multiple comparison corrections, depending on the data set, using GraphPad Prism 10.2.0. Data from in vivo animal experiments were analyzed by one-way or two-way ANOVA followed by Bonferroni correction using GraphPad Prism. For all tests, *p* < 0.05 was considered to be significant.

## Electronic supplementary material

Below is the link to the electronic supplementary material.

Supplementary Material 1

## Data Availability

The data supporting the findings of this study are included in the article and its supplementary information.
