# Supplementary material for: Cryoelectrospun elastin-alginate scaffolds as potential cell delivery vehicles for mesenchymal stromal cell therapy
Source: Sci Rep. 2025 Jun 5;15:19847. doi: 10.1038/s41598-025-03822-x (PMC12141519; doi:10.1038/s41598-025-03822-x)
Supplement: Supplementary file 1 — Supplementary Material 1 [file 41598_2025_3822_MOESM1_ESM.pdf]

SUPPLEMENTARY INFORMATION

Cryoelectrospun elastin-alginate scaffolds as potential cell delivery vehicles for mesenchymal stromal cell therapy

Pujhitha Ramesh, Rafael Pena, Jennifer M. Morrissey, Nicholas Moskwa, Kate Tubbesing, Xulang Zhang, Deirdre Nelson, James Castracane, Alexander Khmaladze, Susan T. Sharfstein, Melinda Larsen, and Yubing Xie

Supplemental Methods

Supplementary Data

Tables S1-S3

Figures S1-S10

Supplemental Methods

1. <u>Cell seeding on scaffolds</u>

1.1. Stromal cell isolation

The embryonic salivary gland was enriched for stromal cells with an MSC-like phenotype. Embryonic day 16 (E16) mouse embryos were harvested. The SMGs were removed and reduced to a mixture of mixed stromal cells using collagenase/hyaluronidase and dispase mechanical dissection. Gravity sedimentation was used to separate the MSCs from the epithelial clusters. The wells were coated with Lipidure to ensure the cells did not stick. The resulting supernatant was enriched for the MSC-like primary E16 salivary mesenchyme cells (SMSCs), where the cells were later collected and seeded on scaffolds at passage 0 (P0). These SMSCs were incubated on the scaffolds for 3 days prior to implantation.

1.2. Well plate preparation for cell seeding and culture

To improve cell attachment efficiency and prevent cells from attaching to the well bottom after cell seeding, round bottom 96-well plates were coated with the ultra-low adhesion polymer Lipidure. Each well was coated thrice by adding 75 µL of 0.64% Lipidure in 96% ethanol to each well, aspirating after 1 minute and air-drying for 15 minutes. After the third coating, the well plate was UV sterilized for 1 hour and air-dried overnight before setting the scaffolds for cell culture. Similarly, 48-well and 24-well plates were coated with Lipidure for cell culture.

1.3. Cell seeding onto scaffolds

SMSCs in DMEM/F12 medium containing 10% FBS and 1% PenStrep were seeded for immunocytochemistry experiments at appropriate cell densities, as detailed in Table S1. To maintain the alginate in its crosslinked form and prevent rapid disintegration of the scaffold, the cell culture medium was supplemented with 25 mM $CaCl_2$, a concentration at which cell culture was not negatively impacted in cryoelectrospun scaffolds (CES). CES and decellularized salivary gland matrices (DSG) were incubated on a rotary shaker at 30 rpm for 2 hours to enhance cell attachment to these 3D scaffolds. After two hours, each well was supplemented with 175 µL of fresh medium, and the well plate was incubated with rotary shaking for another 22 hours to increase the cell attachment efficiency.

SMSCs on Matrigel were seeded and grown in static culture since cells are in a crosslinked suspension, thereby not requiring enhanced cell attachment through rotary culture. All scaffolds were incubated in a humidified incubator at 37 °C and 5% CO_2. For PCR analysis, the number of cells seeded was increased to 75,000 cells/scaffold for cryoelectrospun scaffolds, decellularized salivary gland matrices and Matrigel for increased mRNA yield.

1.4. Cell attachment efficiency analysis

SMSCs were seeded at a set concentration (see Table S1) and allowed to attach to each scaffold for 24 hours. After 24 hours, unattached cells were aspirated by gently pipetting. The scaffold was rinsed gently in cell culture media to collect

any remaining unattached cells. The scaffold was moved to a new well, and any cells attached to the bottom of the well were trypsinized, neutralized, and added to the suspension of cells not attached to the scaffold. The cell suspension was centrifuged at 450g for 5 minutes, resuspended in cell culture media, and the number of cells was counted. Cell attachment efficiency was determined by subtracting the number of cells not attached to the scaffold from the number of cells seeded (Equation 1).

$$Cell\ attachment\ efficiency\ (\%) = \frac{No.of\ cells\ seeded - No.of\ cells\ not\ attached}{No.of\ cells\ seeded} * 100 \qquad (1)$$

2. Immunocytochemistry analysis of marker expression

2.1. Immunocytochemistry and confocal imaging

Cell culture samples were fixed in 4% paraformaldehyde (Thermo Fisher Scientific) in 5% (w/v) sucrose (Sigma-Aldrich), 0.6X PBS (Thermo Fisher Scientific) for 15 minutes, permeabilized with 0.1% Triton X-100 (Sigma-Aldrich) in 1X PBS for 15 min, blocked with 20% donkey serum (Jackson ImmunoResearch Laboratories, West Grove, PA)/3% bovine serum albumin (Thermo Fisher Scientific) in wash buffer (0.9% NaCl-50 mM $CaCl_2$ in deionized water) for 2 hours at room temperature, incubated with primary antibodies at 4 °C overnight, followed by incubation with 4′,6-diamidino-2-phenylindole (DAPI, Sigma-Aldrich) and secondary antibodies at room temperature for 2 hours. Primary E16 mesenchyme cells were immunostained for mesenchymal markers (PDGFRα/CD140a and vimentin) and myofibroblast markers (calponin (CNN1) and α-smooth muscle actin (α-SMA)) while myofibroblasts alone or co-cultures of myofibroblasts and primary mesenchyme cells were stained for PDGFRα, CNN1, and α-SMA. Antibody details and concentrations used are detailed in Table S2. All cells were co-stained with DAPI to reveal the nuclei within the total cell population. Samples were then mounted using a glycerol-based mounting medium [1] for imaging. Confocal imaging was performed using a Leica SP5 confocal laser scanning microscope (Leica Microsystems, Mannheim, Germany).

2.2. Co-immunostaining cell-scaffold constructs with PDGFRα and vimentin antibodies

Samples to be co-immunostained for PDGFRα and vimentin were first stained with primary antibodies and their respective secondary antibodies as per the protocol detailed in the immunocytochemistry method in section 3.1. Afterward, the samples were blocked with 20% rabbit serum-3% bovine serum albumin in wash buffer (0.9% NaCl-50 mM $CaCl_2$ in deionized water) for 2 hours at room temperature and then incubated with AF488-vimentin direct conjugate antibody with gentle rocking at 45 rpm at room temperature for 2 hours. All cells were co-stained with DAPI (Sigma-Aldrich) to reveal the nuclei within the total cell population. Samples were then mounted using a glycerol-based mounting medium181 for imaging. Confocal imaging was performed using a Leica SP5 confocal laser scanning microscope (Leica Microsystems, Mannheim, Germany).

3. qPCR analysis

SMSCs were grown on cryoelectrospun scaffolds, decellularized salivary gland matrices, and Matrigel with and without TGF-β1 and/or FGF2 for 1 or 7 days. For PCR analysis, three or four samples of each scaffold type and experimental condition were pooled to extract enough RNA using an RNeasy Micro kit (Qiagen, Germantown, MD). Lysis buffer was added to samples, followed by vigorous pipetting and vortexing to break apart the scaffold and lyse all cells. RNA was then isolated as per the protocol specified by Qiagen. RNA was measured by the QubitTM fluorometer (Invitrogen, Waltham, MA), and cDNA was synthesized using Maxima H minus First Strand cDNA synthesis kit (Thermo Fisher Scientific). The concentration of the synthesized cDNA was also measured on the QubitTM fluorometer. Primers (Table S3) for housekeeping gene (UBC), mesenchymal markers (PDGFRα and vimentin), and myofibroblast marker (α-SMA) from Integrated DNA Technologies (Coralville, IA), cDNA, and SYBR Green PCR master mix (Thermo Fisher Scientific) were added to the respective wells of a 0.1 mL 96 well PCR plate and assayed on the StepOne Plus Real-Time PCR system (Applied Biosystems, Waltham, MA).

4. Quantification of LIVE/DEAD assay

The 3D image ('lif' image) obtained on the confocal microscope was opened in the Imaris software (Oxford Instrumentation) and converted to an '.ims' file. The image was opened in surpass view, and two 'Spots' objects were created for counting live cells in the green channel and dead cells in the red channel. The spot size was set to 15 µm to identify the cells in each channel. The number of cells in each channel were exported from the 'Statistics' tab. The percentage of live (in green channel) and dead cells (in red channel) was computed for all scaffolds for a minimum of 3 replicates.

5. Intensity quantification of 3D images using Imaris

3D images (z-stacks) in '.lif' format obtained from confocal imaging on the Leica SP5 confocal microscope were converted to '.ims' files on the Imaris 9.6.1 (Oxford Instruments, Tubney Woods, Abingdon, UK). The number of nuclei in the 3D image was quantified by opening the file in surpass view, creating a new surface, setting the smoothing surface grain size to ~0.6 µm, selecting background subtraction, and manually setting the threshold value to select all nuclei. Artifacts that were not cell nuclei were excluded using the filter option and filtering based on quality, area, or sphericity. The individual nuclei were viewed in a grid pattern in the vantage view using the 'gallery' option to count the number of nuclei. Merged nuclei were either segmented in the surpass view by enabling 'split touching objects' and setting the seeding diameter to > 8 µm or manually counting merged nuclei based on the number of merged nuclei. To quantify the sum of the intensity of each voxel for each channel in the region of interest (ROI), a new surface was created again in surpass view. The channel with the most robust protein expression was used to identify the ROI for intensity sum calculation. The smoothing surface grain size was set to ~1.5 µm, and the threshold value was manually adjusted to select the ROI. Artifacts not included in the ROI were excluded by using the filter option and filtering based on quality and area. The intensity sum values for each channel in the ROI were exported to an Excel file in the vantage view. The protein expression levels in each image were quantified by normalizing the intensity sum for each channel to the number of nuclei in the 3D image (Figure S1). Fold changes in protein expression levels were determined by normalizing expression levels from day 7 to day 1.

6. <u>Tissue preparation and cryopreservation</u>

Salivary glands from C57 BL/6J mice were fixed in 4% paraformaldehyde for 2 hours at 4 °C and washed thrice in 1X phosphate buffered saline (PBS). The glands underwent a series of sucrose gradient concentrations, going from 5% to 10% and then to 15%. The glands remained in each sucrose concentration for one hour at 4°C before being transferred to a higher concentration. After the 15% concentration, the glands sat in 30% sucrose concentration overnight at 4°C before being transferred into 15% sucrose concentration (with 50% OCT tissue freezing medium (Electron Microscopy Sciences, Hatfield, PA) overnight at 4°C. The glands were placed in cryomolds and the cryomolds are filled with tissue freezing media. The glands were frozen indirectly with liquid nitrogen and stored at -80°C before cryosectioning. All subsequent sample processing, image capturing, and quantitative analysis were performed blinded with reference to the unique identifiers from the ear punches.

7. <u>Cryosectioning of salivary glands</u>

Samples were collected by serial sectioning 10-µm sections and assembling 6 sections per slide. One section was collected on one slide before moving onto the next slide and placing the serial section on that slide. This process was repeated until 27 slides contained a section. The process was repeated until all 27 slides contained six sections, representing the entire gland. Sections were dried for 30 minutes at room temperature and then stored at -80°C before staining.

8. <u>Immumohistochemistry analysis of cryosection slides</u>

Slides were fixed in 4% paraformaldehyde for 18 minutes, and then washed two times in 1x phosphate buffered saline (PBS) for 3-5 minutes. Slides were then permeabilized in 0.5% 100X Triton for 18 minutes, before being washed two more times with PBS for 3-5 minutes. Slides were blocked for one hour at room temperature using 3% bovine serum albumin (BSA) and 10% donkey serum. Afterwards, slides were incubated with primary antibody against macrophage marker F4/80, M2 marker CD206 (direct conjugate), or lymphocytic B-cell marker CD45R for evaluation of inflammatory response, or against PDGFRα, PDGFRβ, vimentin, α-SMA (direct conjugate), or calponin for expression of mesenchymal and myofibroblast markers (see Table S2) for one hour at room temperature and then washed three times with 1X PBS for 3-5 minutes. Slides were incubated with secondary antibody (See Table S2) for one hour at room temperature and then washed two times with 1X PBS for 3-5 minutes. Slides were then incubated with direct conjugate for one hour at room temperature and then washed with 1X PBS two times for 3-5 minutes. Afterwards, slides were incubated in DAPI for 10 minutes and washed two final times in 1X PBS for 3-5 minutes. Slides were then mounted with a glycerol based mounting media.

9. <u>Quantification of cryosection slides</u>

Quantification was performed on sections from similar tissue depths. Only the SMG was included in the quantification. The fibrotic region was compared between sections. To compare a non-fibrotic area with the fibrotic area, the region of interest (ROI) was slightly shifted from the fibrotic region. Images were quantified using FIJI, ImageJ. To quantify DAPI & tissue, the images were thresholded to capture as much of the positive area without over-capturing the images. To quantify the area of protein expression positive for the antibody activity, the background was subtracted, and the images were thresholded. The positive pixel area was normalized to total area of the ROI then multiplied by 100% to get a percentage of how much of the area was positive for the antibody relative to the area of the ROI. Similarly, to quantify PSR area, the images were thresholded to capture the positive stain and divided by the total area of the fibrotic region.

10. Identification of fibrotic and non-fibrotic regions in cryosections:
The isolated glands were weighed relative to total mouse weight (Figure S9A). Cryopreserved SMGs were sectioned, and sections were stained with 4',6-diamidino-2-phenylindole (DAPI) to identify the nuclei of the total cell population and with picrosirius red (PSR) to reveal collagen fibers and to identify the local region of fibrosis; regions with higher nuclear density, increased PSR staining and aberrant morphology of nuclear patterning and tissue, were identified as fibrotic compared to the rest of the gland (Figure S9B-E).

Supplementary Reference

[1] K. Valnes, P. Brandtzaeg, Retardation of immunofluorescence fading during microscopy, J. Histochem. Cytochem. 33 (1985) 755–761. https://doi.org/10.1177/33.8.3926864.

Supplementary Data

Optimization of Cell Seeding on Elastin-Alginate Cryoelectrospun Scaffolds (CES)

To ensure high cell attachment to the three-dimensional CES, we tested the effect of cell seeding densities and rotary shaking by seeding SMSCs at 50,000, 100,000 or 150,000 cells per CES with and without mild rotary shaking at 30 rpm for the first 24 hours in ultra-low adhesion polymer-coated, round-bottom wells in a 96-well plate. Rotary shaking improved cell attachment in CES in the groups where 50,000 and 100,000 cells were seeded per scaffold and reduced the variability in cell attachment compared to static culture (Figure S2A). Cells seeded at 100,000 and 150,000 cells per CES with rotary shaking attached heterogeneously as massive clusters to CES (Figure S2C, D), whereas cells seeded at 50,000 cells/CES homogenously attached to CES (Figure S2B); hence for all experiments, cells were seeded at 50,000 cells per CES and DSG in a 96-well plate with gentle rotary shaking at 30 rpm for the first 24 hours. After the first 24 hours of cell seeding on these scaffolds, cell-scaffold constructs were transferred to a 24-well plate with 300 µL media in each well for improved oxygen diffusion and cell viability, relative to 50% viability in a 48-well plate (Figure S3). We observed that the primary E16 mesenchyme cells were barely attached to NF, and negligible numbers of cells remained on the scaffold on day 7 (Figure S4), consistent with our previous work using NIH 3T3 fibroblasts grown in CES.

Supplementary Tables

Table S1. Cell seeding and culture strategy for improved cell attachment and viability

**Scaffold**	**Cells seeded**	**Cell attachment (first 24 hours)**	
		**Culture dish**	**Culture strategy**
**Cryoelectrospun scaffolds (CES)**	50,000 cells/25 µL media	Round-bottom Lipidure coated 96-well plate	Rotary culture
**Decellularized salivary gland matrix (DSG)**	50,000 cells/25 µL media	Round-bottom Lipidure coated 96-well plate	Rotary culture
**Matrigel**	50,000 cells/10 µL cell-Matrigel suspension in 180 µL media	0.1 µm Nucleopore filter in Mattek dish	Static culture

Table S2. Antibodies used for immunocytochemistry and immunohistochemistry analysis

Primary Antibody	Host Species	Company	Catalog No.	Lot No.	Dilution
**Cells (immunocytochemistry)**					
CD140a (PDGFRα)	Rat	Thermo Fisher	12-1401-81	-	1:200
CD140b	Rabbit	Abcam	ab32570	-	1:200
CD73	Rat	Thermo Fisher	14-0731-81	-	1:100
CD105	Rat	Thermo Fisher	14-1051-81	-	1:100
Vimentin (Clone LN6)	Mouse	Sigma	V2258	-	1:2000
**Cell-scaffold constructs (immunocytochemistry)**					
CD140a (PDGFRα)	Rat	ThermoFisher Scientific	14-1401-81	2015727	1:100
Calponin 1 (CNN1)	Rabbit	Abcam	ab46794	-	1:600
α-SMA	Mouse	Sigma Aldrich	A5228	-	1:1000
**Direct Conjugate**					
Vimentin Alexa Fluor® 488	Rabbit	Cell Signaling Technology	9854S	12	1:200
**Salivary gland cryosections (immunohistochemistry)**					
F4/80	Rat	BioLegends	AB_893504	-	1:200
CD45R	Rat	BioRad	RA3-6B2	-	1:200
PDGFR-α	Goat	R&D Systems	AF1062-SP	-	1:100
PDGFR-β	Rat	Invitrogen	14-1402-82	2388135	1:100
Calponin 1	Rabbit	Abcam	AB46794	-	1:100
Vimentin	Rabbit	Cell Signaling	5741S	-	1:100
**Direct Conjugate**					
CD206 – AF647	Rat	BioLegends	141711	-	1:100
α-SMA Alexa Fluor™ 488	Mouse	Thermo Fisher Invitrogen	53-9760-80	-	1:200
**Secondary Antibody**	**Species**	**Company**	**Catalog No.**	**Lot No.**	**Dilution**
Alexa Fluor® 488 AffiniPure F(ab')$_2$ Fragment IgG, Rabbit	Host: Donkey<br>Target: Anti-Rabbit	Jackson ImmunoResearch	711-226-152	132511	1:250
Cy™3 AffiniPure F(ab')$_2$ Fragment IgG (H+L), Rat	Host: Donkey<br>Target: Anti-Rat	Jackson ImmunoResearch	712-166-153	139421	1:500
Cy™3 AffiniPure F(ab')$_2$ Fragment IgG (H+L), Goat	Host: Donkey<br>Target: Anti-Goat	Jackson ImmunoResearch	707-166-147	107019	1:500
Alexa Fluor® 647 AffiniPure F(ab')$_2$ Fragment IgG (H+L), Rabbit	Host: Donkey<br>Target: Anti-Rabbit	Jackson ImmunoResearch	711-606-152	125599	1:500
Alexa Fluor® 647 AffiniPure F(ab')$_2$ Fragment IgM, μ chain specific	Host: Donkey<br>Target: Anti-Mouse-μ chain specific	Jackson ImmunoResearch	715-606-020	135520	1:250
Alexa Fluor® 647 AffiniPure F(ab')$_2$ Fragment IgG (H+L), Rat	Host: Donkey<br>Target: Anti-Rat	Jackson ImmunoResearch	712-606-153	112853	1:500

Table S3. Primers used for PCR analysis

Primer	Direction	Gene Sequence
UBC	Forward	ACAGACGTACCTTCCTCACCA
	Reverse	CCCCATCACACCCAAGAACAA
Vimentin	Forward	CGCCCTCATTCCCTTGTTGC
	Reverse	GGTAGGAGGACGAGGACACAG
αSMA	Forward	AATGTCCCCGCCATGTATGT
	Reverse	TTTCGTGGATGCCCGCTG
PDGFRα	Forward	CACAATAACGGGAGGCTGGT
	Reverse	CACCTCCACCACGAACTCTC

Supplementary Figures

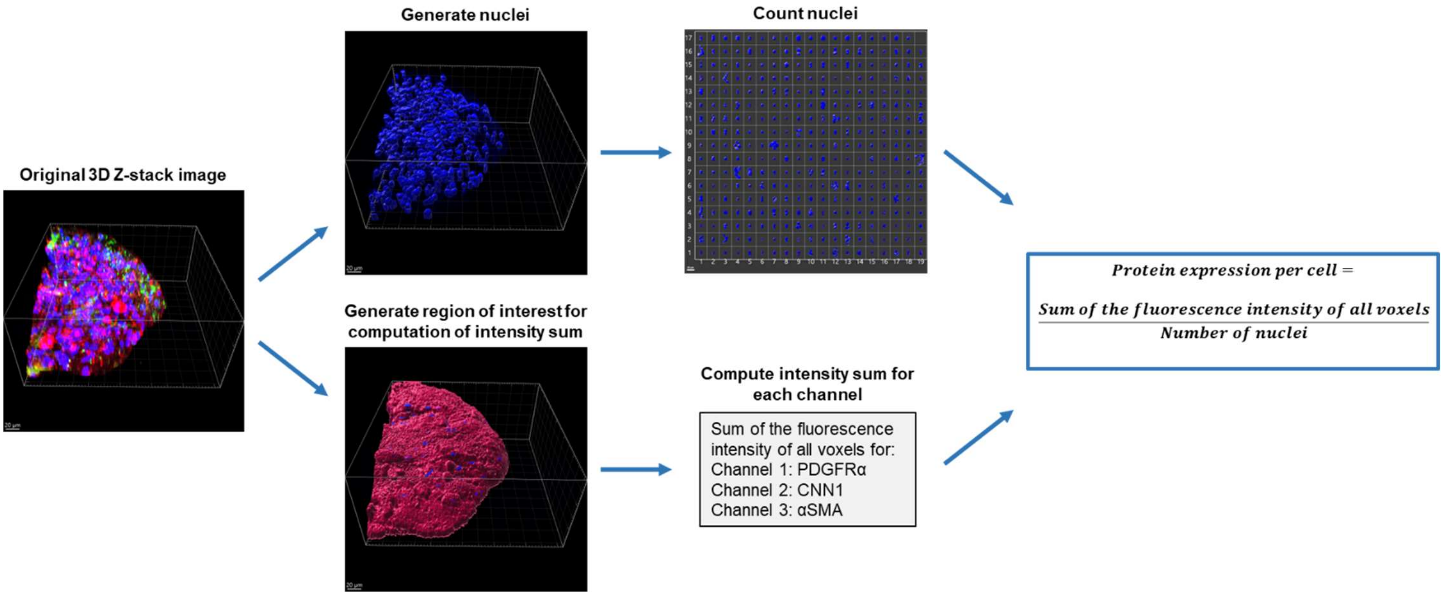

Figure S1. Schematic diagram detailing the protocol for quantification of protein expression per cell from 3D confocal images.

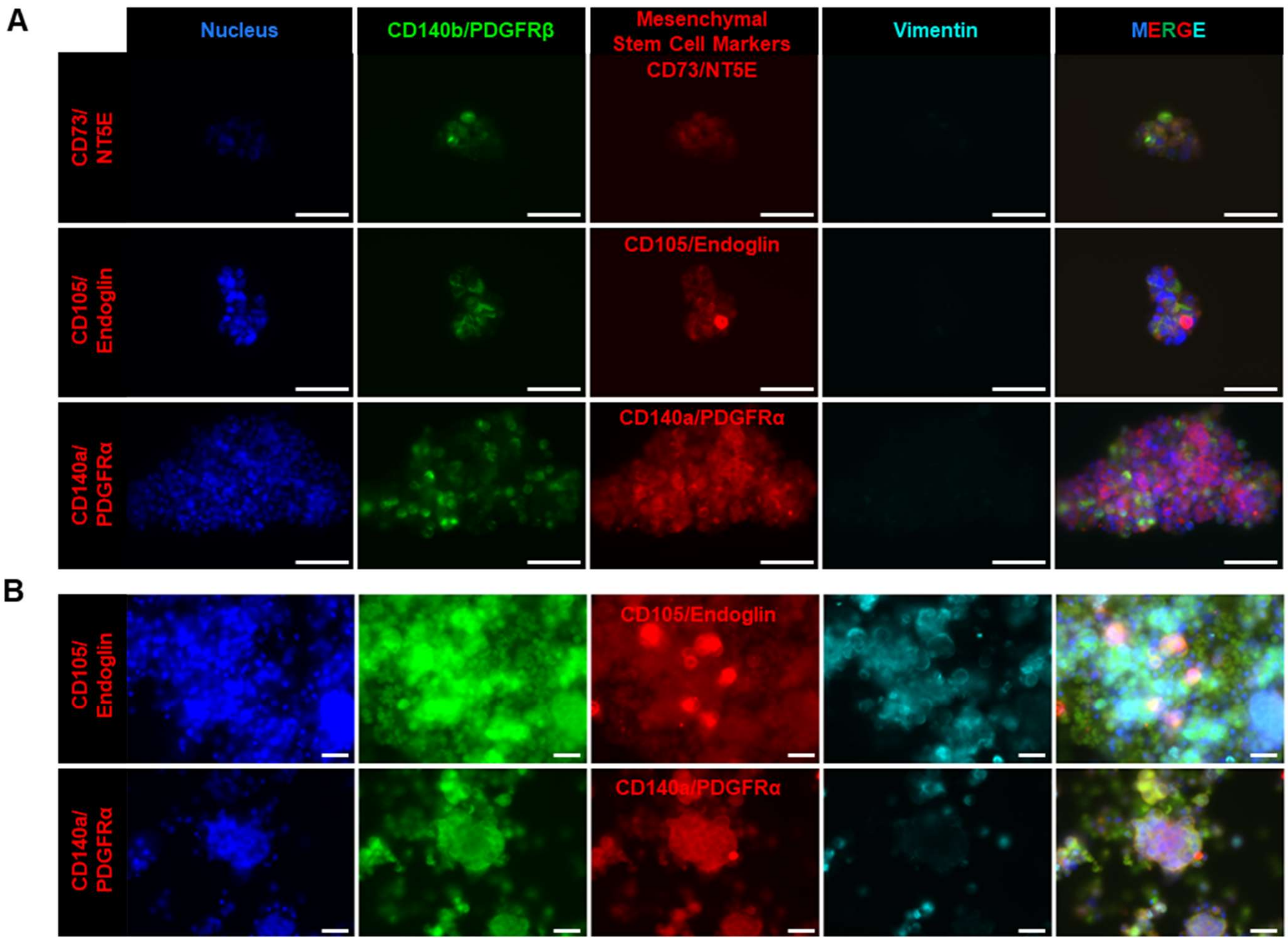

Figure S2. Mesenchymal stromal marker expression in SMSCs isolated from embryonic salivary tissue and grown for A) 0 hours in suspension and B) 4 hours after cells adhered to tissue culture plastic. Fluorescence microscopy images showing expression of mesenchymal stem cell markers CD140a, CD73 and CD105 in red, stromal markers CD140b in green and vimentin in teal. Scale bar = 50 μm.

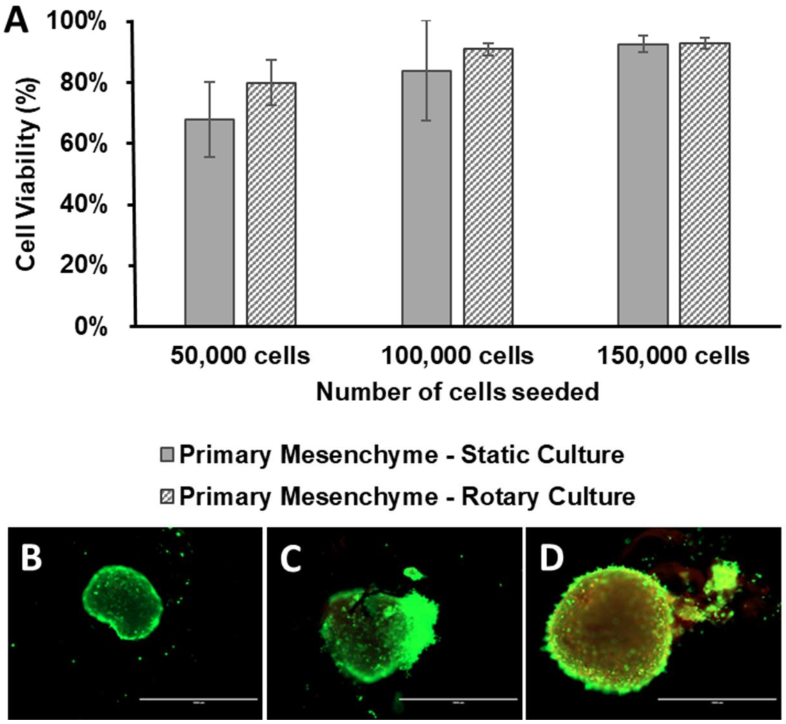

Figure S3. Effect of cell seeding density and dynamic culture on SMSC growth in CES. A) Cell seeding efficiency of static vs. rotary culture (at 30 rpm for the first 24 hours only) of SMSCs seeded onto elastin-alginate cryoelectrospun scaffolds (CES). 50,000-150,000 cells in 25 µL cell culture media supplemented with 25 mM $CaCl_2$ were seeded in each scaffold in a Lipidure-coated, non-adhesive 96-well plate and supplemented with 175 µL of the same medium composition after 2 hours. Rotary culture improves cell attachment and reduces variability in cell attachment. Fluorescent microscopy images of Live/Dead staining of SMSCs cultured with rotary shaking for the first 24 hours at 30 rpm attach homogenously as single cells when seeded at B) 50,000 cells/scaffold, but they attach heterogeneously as clusters when seeded at C) 100,000 cells/scaffold and D) 150,000 cells/scaffold. Scale bar = 1000 µm.

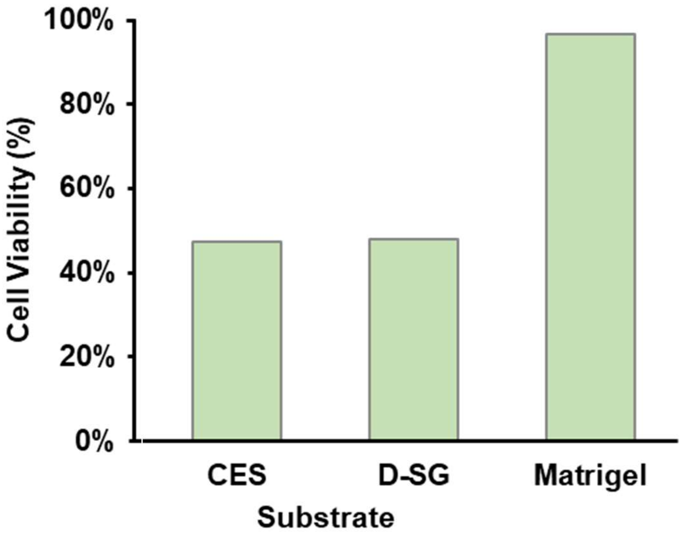

Figure S4. Viability of SMSCs grown for up to 7 days in a 48-well plate. Quantification of cell viability on elastin-alginate cryoelectrospun scaffolds (CES), decellularized salivary gland matrices (DSG) and Matrigel using Imaris after 7 days of cell growth, reveals that cells on CES and DSG have low cell viability compared to Matrigel when cultured in a 48-well plate due to poor nutrient diffusion into the 3D tissues.

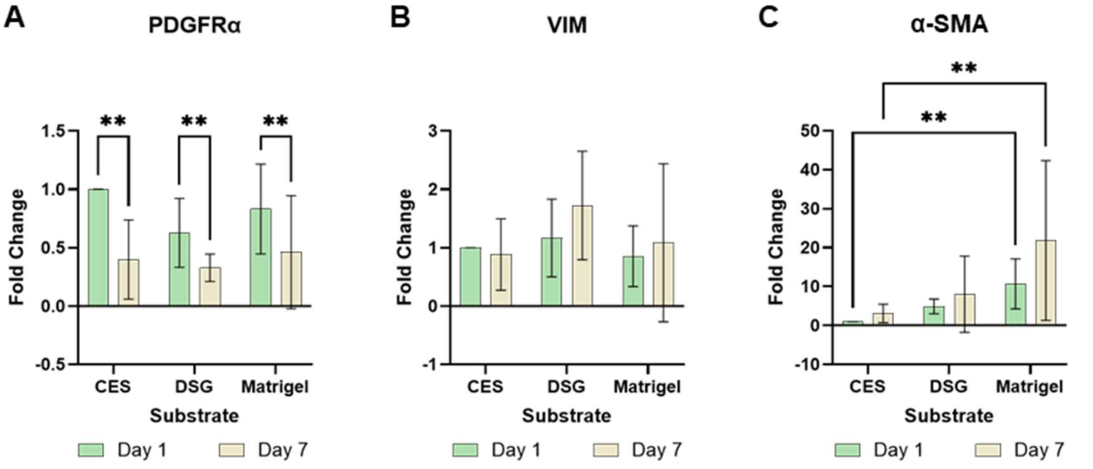

Figure S5. qPCR gene expression analysis of stromal and myofibroblast markers in 3D cultured SMSCs in cryoelectrospun scaffolds (CES), decellularized salivary gland matrices (DSG) and Matrigel. mRNA expression of (A) PDGFRα and (B) vimentin confirms maintenance of healthy stromal phenotype in CES comparable to DSG. (C) mRNA expression of α-SMA corroborates the repression of fibrotic markers in CES comparable to DSG, which are significantly lower than Matrigel on day 1 and day 7, respectively. Statistical analysis was performed by two-way ANOVA with uncorrected Fisher's LSD for multiple comparisons. **, $p<0.01$.

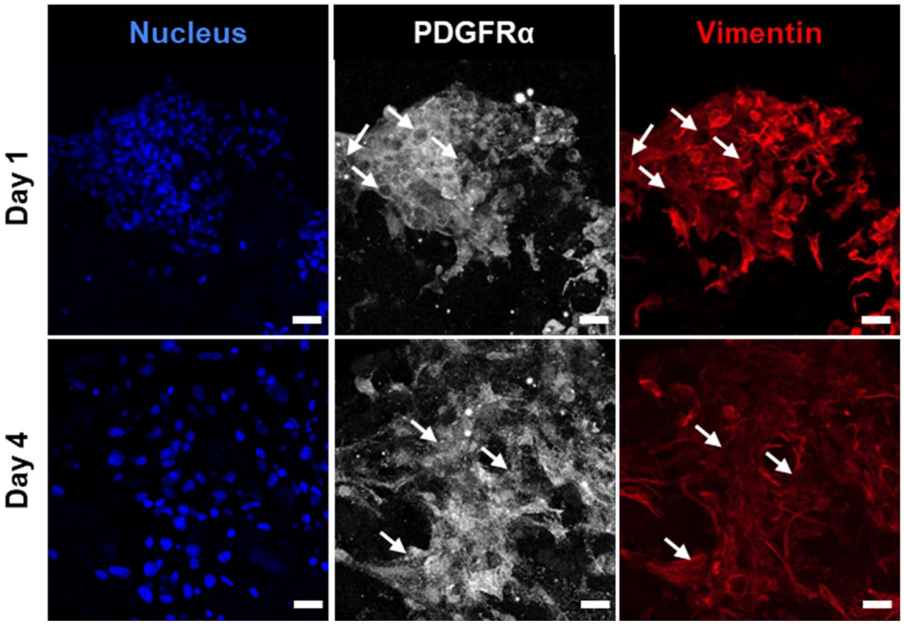

Figure S6. SMSCs cultured in elastin-alginate cryoelectrospun scaffolds (CES) are relatively more spread out on day 4 than on day 1, as observed by membrane-localized PDGFRα (white) and cytoskeletal vimentin (red). Blue, DAPI-stained nuclei to reveal the total cell population. Arrows in top panel indicate rounded morphology of cells whereas in bottom panel indicate spread out morphology of cells. Scale bar = 20 μm.

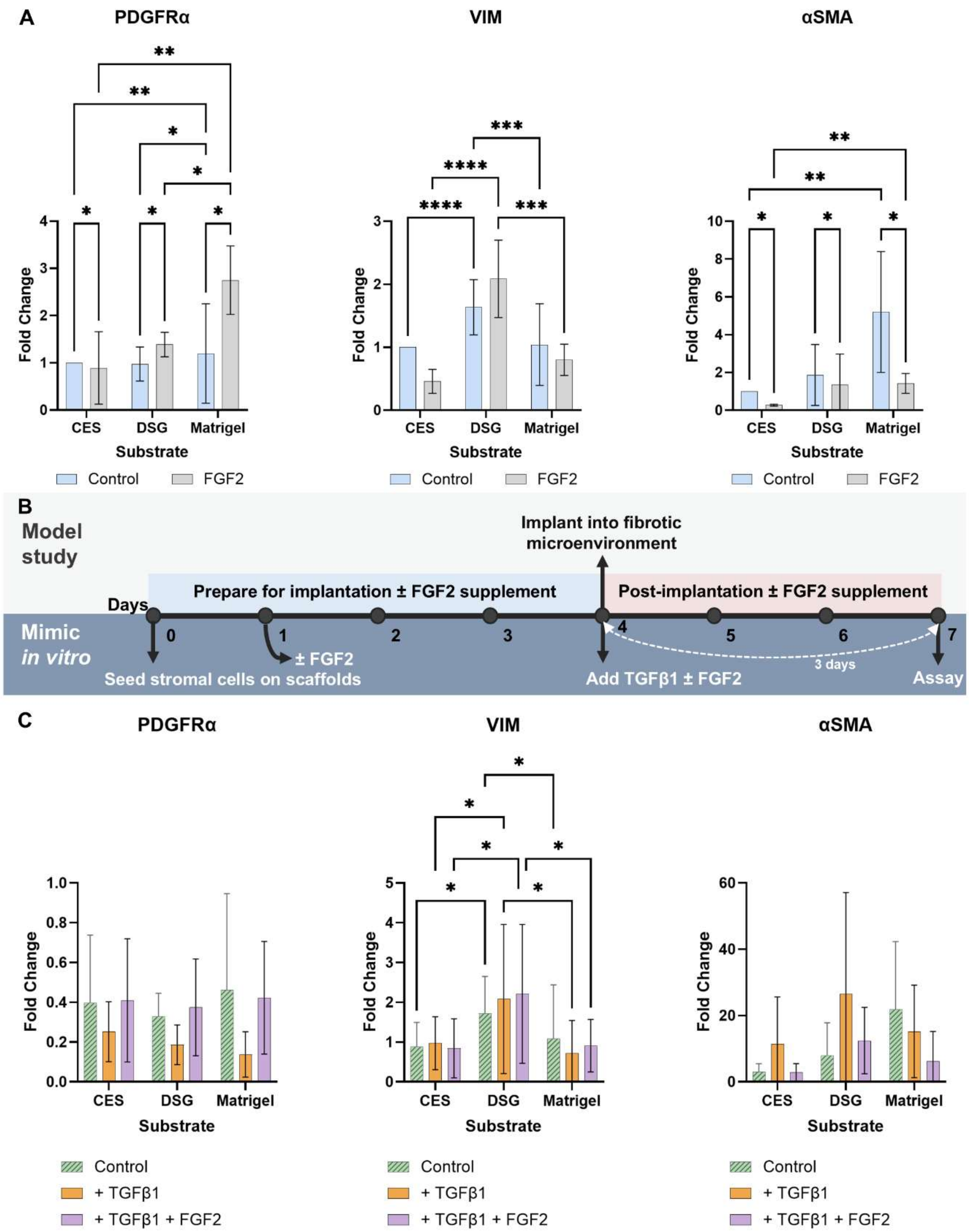

Figure S7. FGF2 fortifies healthy stromal marker expression in cryoelectrospun scaffolds (CES). qPCR gene expression analysis of stromal and myofibroblast markers in 3D cultured SMSCs in CES, decellularized salivary gland matrices (DSG) and Matrigel with and without FGF2 and/or TGF-β1 stimulation, after 7 days in culture. A) mRNA level expression of PDGFRα, vimentin and α-SMA indicates that FGF2 maintains healthy stromal phenotype and represses fibrotic phenotype in CES and Matrigel after 7 days in culture. B) Schematic of *in vitro* experiment design to recapitulate and study stromal response to implantation into a fibrotic environment, which mimics the cellular cue using myofibroblasts and biological cue using pro-fibrotic TGF-β1, and effects of FGF2 stimulation on modulating fibrotic marker expression. C) Gene expression analysis comparing the effect of FGF2 stimulation on the expression of PDGFRα, vimentin and α-SMA expression in SMSCs in CES vs. DSG vs. Matrigel in a TGF-β1-induced fibrotic microenvironment, on day 7. Statistical analysis was performed by two-way ANOVA with uncorrected Fisher's LSD for multiple comparisons. *$p<0.05$, **$p<0.01$, ***$p<0.001$. ****$p<0.0001$.

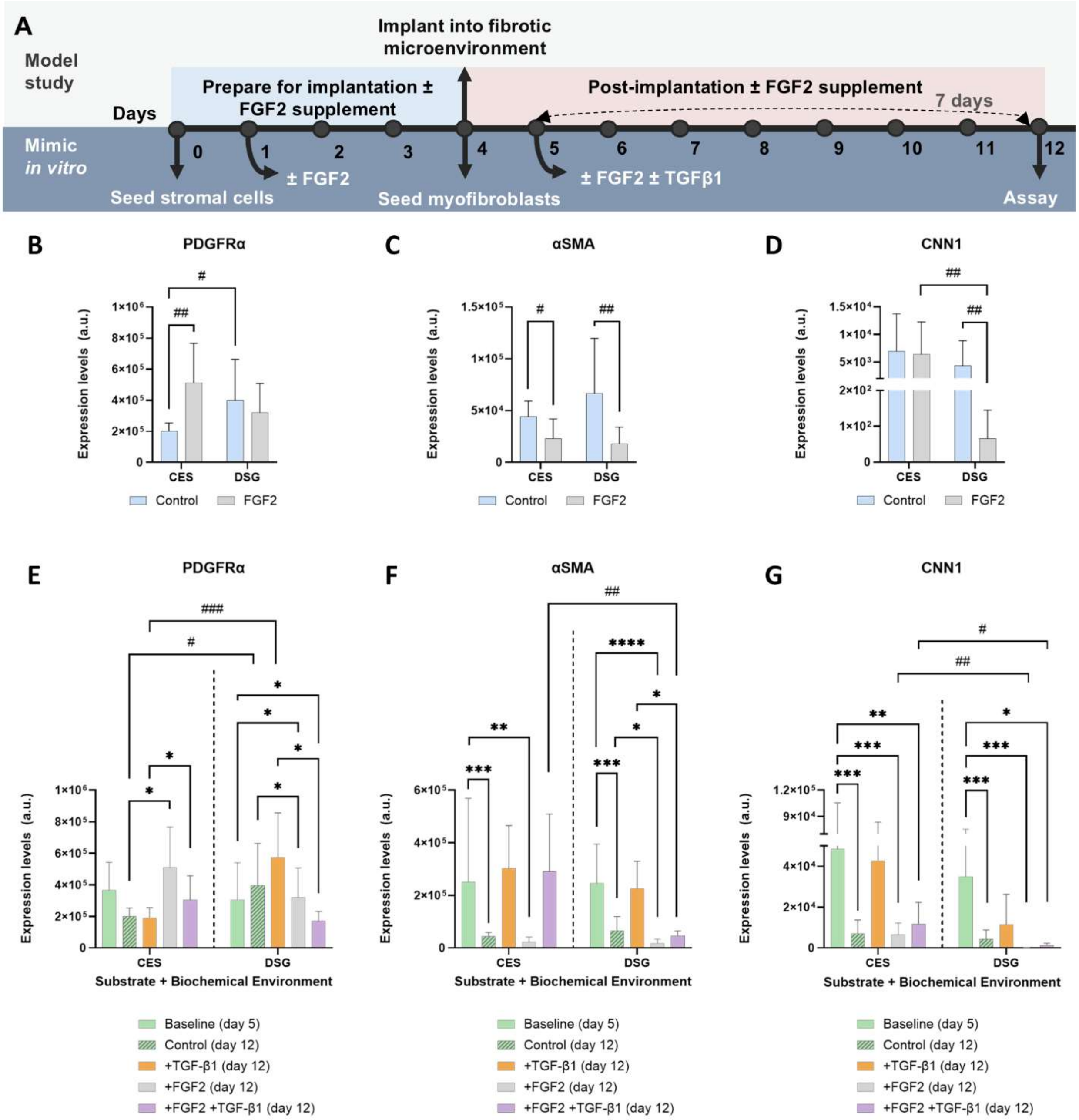

Figure S8. FGF2 supplementation potentiates the suppression of fibrotic markers in SMSC and myofibroblast cocultures in *in vitro* fibrosis model. When co-stimulated with TGF-β1, the anti-fibrotic effects of SMSCs is moderately rescued by FGF2 in CES, and more potently rescued in DSG. A) Schematic of *in vitro* experiment designed to mimic implantation into a fibrotic environment and interaction of stromal cells ± FGF2 with myofibroblasts ± TGF-β1, assayed on day 12. Fluorescence intensity-based quantitative analysis of B) PDGFRα, C) α-SMA and D) CNN1 expression in myofibroblasts and SMSCs after 7 days of co-culture in the absence or presence of FGF2 and in the absence of TGF-β1. For comparisons between substrates or treatment groups, statistical analysis was performed using multiple unpaired t-tests without correction for multiple comparisons (p values denoted by #). Fluorescence intensity-based quantitative analysis of E) PDGFRα, F) α-SMA and G) CNN1 expression in myofibroblasts and SMSCs after one day of co-culture on day 5 (Baseline Day 5), and after 7 days of co-culture in the absence of FGF2 and TGF-β1(Control Day 12), in the presence of TGF-β1 stimulation alone (+ TGF-β1 Day 12), in the presence of FGF2 stimulation alone (+ FGF2 Day 12) and in the presence of both FGF2 and TGF-β1 stimulation (+FGF2 + TGF-β1 Day 12). Statistical analysis was performed by two-way ANOVA with uncorrected Fisher's LSD for multiple comparisons between the different treatment groups within the same substrate (p values denoted by *). For comparisons between substrates, statistical analysis was performed using multiple unpaired t-tests without correction for multiple comparisons (p values denoted by #). *,#p<0.05, **,##p<0.01, ***,###p<0.001,****p<0.0001.

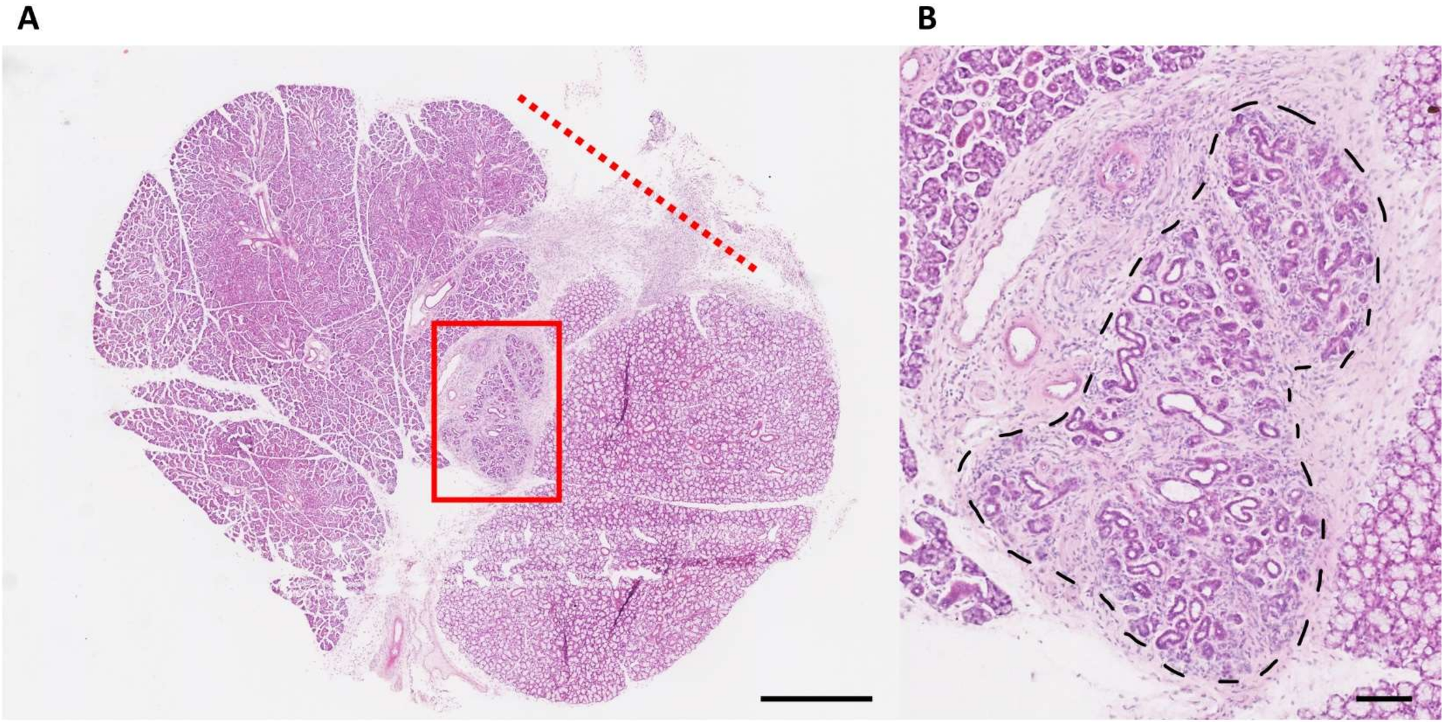

Figure S9. Analysis of tissue morphology of salivary gland after resection. A) Hematoxylin & eosin staining performed on an example section of a resected salivary gland. The local fibrotic region is shown in the red boxes; red dashed lines = gland resection line. Scale bars = 100 µm. B) Close-up of the local fibrotic region (surrounded in the black dashed lines). Abnormal tissue morphology is detected in the fibrotic region compared to the surrounding non-fibrotic tissue. Scale bar = 50 µm. SLG = Sublingual Gland, SMG = Submandibular Gland.

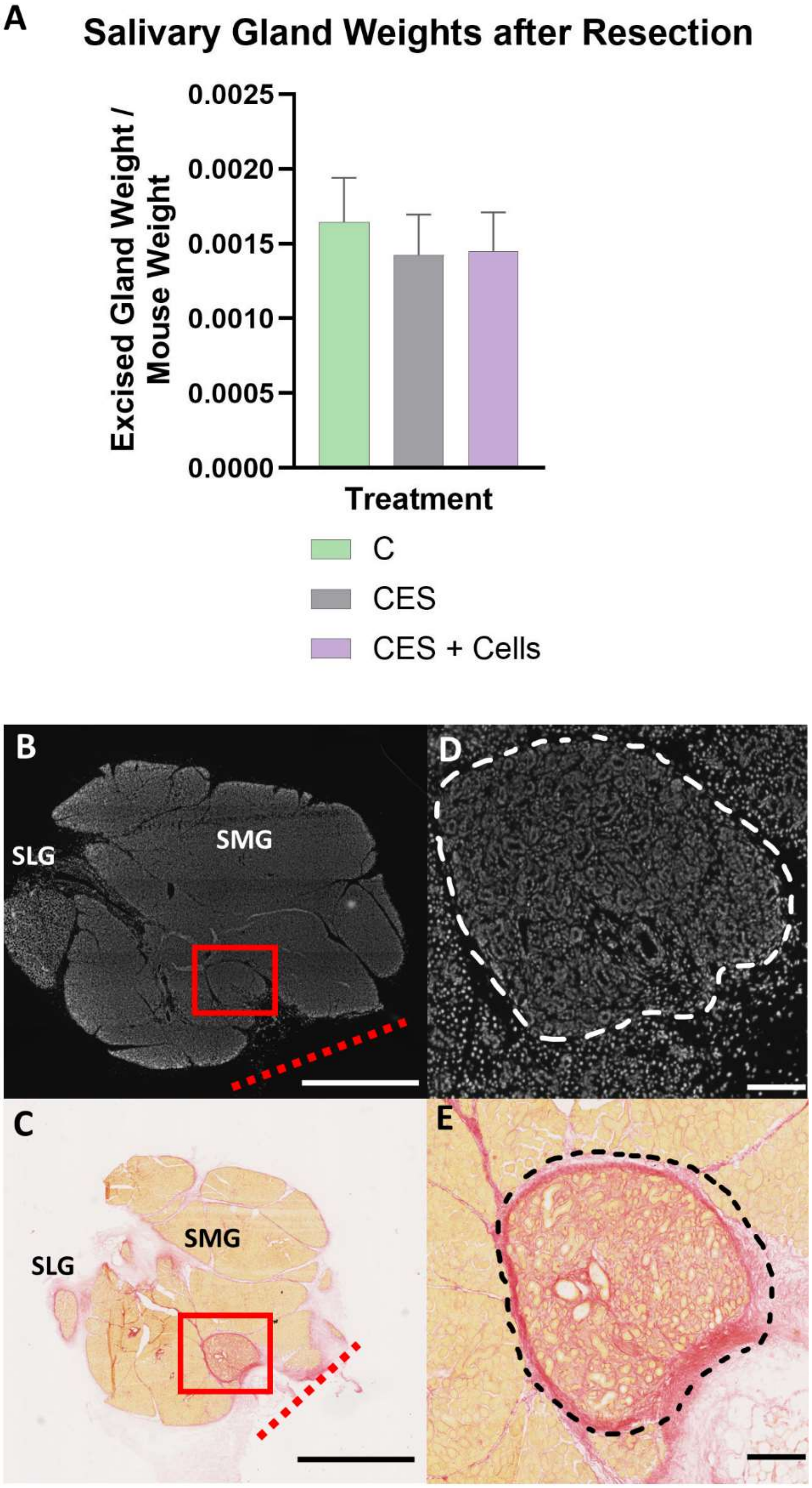

Figure S10. Identifying fibrotic regions and gland weights after surgery. A) Measurements of gland weights normalized to mouse weight after gland resection alone (C) or resection plus implantation of CES without (CES) or CES with SMSCs (CES+Cells) show no significant changes with N= 10 mice for each. Statistical analysis: One-way ANOVA with Bonferroni correction, ns = non-significant. Global image of B) DAPI and C) picrosirius red staining performed on an example section of a resected salivary gland. The local fibrotic region is shown in the red boxes; red dashed lines = gland resection line. Scale bars = 100μm. Close-up of the localized fibrotic region that exhibits a higher nuclear density than the surrounding non-fibrotic region D) in DAPI (dashed white lines) and that exhibits increased fibrosis E) detected with picrosirius red (dashed black lines). Scale bar = 50 μm. SLG = Sublingual Gland, SMG = Submandibular Gland.
